# Four new *Cyclopina* (Copepoda, Cyclopinidae) from South Korea

**DOI:** 10.3897/zookeys.992.54856

**Published:** 2020-11-12

**Authors:** Tomislav Karanovic

**Affiliations:** 1 Hanyang University, College of Natural Sciences, Department of Life Science, Seoul 04763, Republic of Korea Hanyang University Seoul South Korea

**Keywords:** Cyclopoida, intertidal zone, meiofauna, new species, stygofauna, taxonomy

## Abstract

Copepods are well studied in South Korea, with the exception of marine non-parasitic cyclopoids, and especially cyclopinids; only three species were found so far here, and only one of them is endemic. A survey of intertidal interstitial faunas from sandy beaches revealed four endemic members of the genus *Cyclopina* Claus, 1863, which represents the first record of the largest cyclopinid genus in South Korea. A detailed study of their morphology revealed numerous differences, including in rarely studied cuticular organs. Some of these micro-characters could easily be homologised and showed little intraspecific variability, which might prove invaluable for matching sexes and reconstructing phylogenetic relationships. *Cyclopina busanensis***sp. nov.** is described from both sexes collected near Busan (South Coast of South Korea), and is most similar to the only congener from Japan: *C. kiraensis* Horomi, 1984. *Cyclopina koreana***sp. nov.** is described from both sexes collected near Gangneung (East Coast), and has no close relatives among currently known species. *Cyclopina curtijeju***sp. nov.** is described from two females from Jeju (off South Coast); it is possibly closely related to *C. smirnovi* Herbst, 1982, but the latter is known from a single male from the Russian Far East. *Cyclopina wido***sp. nov.** is described from both sexes from Wido (West Coast), and shows numerous reductions in segmentation and armature of appendages, most of them probably a consequence of its diminutive size. A table of 26 discrete and continuous characters commonly used in the taxonomy of this group is provided for 48 valid species and subspecies of *Cyclopina*.

## Introduction

Marine cyclopoids, and especially cyclopinids, are poorly studied globally because their diversity is highest in marginal habitats, such as intertidal interstitial and anchialine caves, or in highly inaccessible abyssal and hadal depths. Only three cyclopinids have been reported so far from Korea: *Cyclopinoides orientalis* Chang, 2011; *Cyclopinopsis deformata* Lee & Chang, 2019; and *Paracyclopina nana* Smirnov, 1935. The first species was described by Chang (2011) from one beach on the East Coast of mainland Korea, one beach on Jeju Island (Korea), and one beach on Tsushima Island (Japan). The second species was described by Lee and Chang (2019) from a shallow littoral (25 m) on the East Coast and intertidal sands on the West Coast of South Korea. The third species was described by Smirnov (1935) from Vladivostok (Russia) and was subsequently reported also from China (Shen 1979), Japan (Ueda et al. 2001), and South Korea (Chang 2009, 2010); interestingly, this cyclopinid has become a model organism for various genomic and physiological studies in recent years (Jeong et al. 2015; Lee et al. 2015, 2017). Chang (2011) also mentioned unidentified specimens belonging to the genus *Cyclopina* Claus, 1863 accompanying *Cyclopinoides orientalis*, but it is unclear if these were collected in South Korea or Japan. It is possible that these specimens are conspecific with one (or more) of the four South Korean species described in this paper, but it is also possible that they belong to *Cyclopina kiraensis* Hiromi, 1984, which is the only species from this genus described so far from Japan and seems to be relatively widely distributed there (Hiromi 1984; Ueda et al. 2001).

Copepods are generally relatively well studied in South Korea, both as free-living forms in marine (Soh et al. 2010; Lee et al. 2012) and freshwater environments (Chang 2009, 2010), as well as parasites of other organisms (Kim 2008). However, utilisation of novel taxonomic methods, such as the study of microstructures (Karanovic and Cho 2012, 2016, 2017; Karanovic and Lee 2012; Karanovic et al. 2013) and DNA (Karanovic and Kim 2014a, b; Karanovic et al. 2014, 2015; Kim et al. 2014), and survey of marginal and previously understudied habitats, such as marine interstitial (Karanovic 2014, 2017; Karanovic et al. 2012a, b; Karanovic and Lee 2016), resulted in numerous recent additions. While most interstitial copepods are harpacticoids (Giere 1993), a recent survey of selected intertidal beaches in South Korea brought to light four new species of *Cyclopina* presented here. There are no published data on how much of the South Korean coastline is sandy, but it is a significant ecosystem without any doubt. South Korea has 12,478 kilometres of coastline along three seas (Pruett and Cimino 2000) and some three-quarters of the world’s ice-free coastlines consist of sandy shores (Brown and McLachlan 2006). Like in most developed economies, this ecosystem is under constant anthropogenic pressure and, being a marginal habitat, is rarely included in protected natural reserves. However, marine interstitial harbours a disproportionate level of biodiversity (Gray 1997; Thrush et al. 2006; Karanovic 2008), which is yet to be fully appreciated and understood (Armonies and Reise 2000; Gray 2002; Zeppilli et al. 2015).

*Cyclopina* is the oldest and type cyclopinid genus, as well as the largest by number of species (Boxshall and Halsey 2004). It was established by Claus (1963), with *C. gracilis* Claus, 1863 as the type species. Approximately 70 other species and subspecies have been described since then, but many of them were subsequently transferred to newly established genera or synonymised. However, this genus still contains more than 30% of cyclopinid species (Boxshall and Halsey 2004; Walter and Boxshall 2020). The most recent key to species and subspecies was provided by Vervoort (1964) and it was based on an earlier one provided by Lindberg (1953). This makes identification of species difficult. A lack of morphological detail in early species descriptions (including nearly half of them described only after one sex) and wide intraspecific variability between highly disjunct populations in some presumably widely distributed species make it impossible to construct a reliable key to species (Karanovic 2008). Also, there are no published lists of characters for all species in the genus; most authors usually comparing new or redescribed species with only a few congeners. Apparent differences in the armature of mouthparts between disjunct populations were often ignored, usually based on suspicion of earlier inadequate descriptions (Jaume and Boxshall 1996), although they proved valuable in distinguishing some Australian congeners (Karanovic 2008). Most *Cyclopina* species, however, have never been recorded and redescribed after their original description, which is arguably the largest problem for the taxonomy of this genus.

Cyclopinid systematics at large is also still in a state of flux (Boxshall and Jaume 2012 and references therein), which is perhaps best illustrated by the fact that Walter and Boxshall (2020) list *Heterocyclopina* Pleşa, 1969 in the family Cyclopinidae and the supposedly closely related genus *Procyclopina* Herbst, 1955 in the family Hemicyclopinidae. Both genera were considered members of the allegedly monophyletic Hemicyclopinidae by Martínez Arbizu (2001a), in addition to *Pseudocyclopina* Lang, 1946 and five other genera. However, the genus *Pseudocyclopina* was considered a member of Cyclopinidae by Elwers et al. (2001), with one of the co-authors being Martínez Arbizu. As noted by Boxshall and Halsey (2004), the phylogenetic analysis presented by Martínez Arbizu (2001a) as a justification for the establishment of the Hemicyclopinidae was not parsimony based and hinged on a single character, which is also present in at least four unrelated genera. Some of the characters used by Martínez Arbizu (2000a, b, 2001a, b, 2006) to define supposedly monophyletic families of cyclopinids were shown to be part of intraspecific variability, and sometimes even asymmetries (Karanovic 2008). A polyphyletic nature of cyclopinids was already suspected by Ho (1986), Ho and Thatcher (1989), and Huys and Boxshall (1991), based on the analysis of morphological characters. It was confirmed by Khodami et al. (2017), based on the analysis of four genes and 205 copepod species. However, the molecular phylogeny presented by Khodami et al. (2017) did not recover monophyly of previously proposed monophyletic families (where they had representatives of more than one genus). The same authors proposed another two new families, each containing a single cyclopinid genus, and one of them a single species. This certainly contributes very little to our understanding of the phylogenetic relationships between cyclopinid genera, but unfortunately, no comprehensive, parsimony-based test of the validity of the new families has yet been carried out (Boxshall and Jaume 2012). It should be noted that subsequent re-analyses of the molecular dataset published by Khodami et al. (2017) failed to reproduce both their topology and branch supports, despite the use of the same methods and software (Mikhailov and Ivanenko 2019a). It is reasonable to conclude that we are still in the early stages of understanding cyclopoid systematics, with wider taxon and character sampling continuing to raise as many questions as they answer (Khodami et al. 2019; but see Mikhailov and Ivanenko 2019b). Validity of many genera is widely disputed among different researches (see, for example, Karanovic 2008; Ivanenko et al. 2019). The fact that nearly 60% of all cyclopinid genera are monotypic (Boxshall and Halsey 2004; Karanovic 2008; Suárez-Morales and Almeyda-Artigas 2015; Walter and Boxshall 2020) clearly indicates that we are not even close to discovering the major extent of their diversity. There is no doubt that we will have to look for alternative characters when trying to reconstruct phylogenetic relationships between cyclopinids. Cuticular organs on somites were recently suggested as suitable micro-characters for reconstructing phylogenetic relationships between some harpacticoid copepods (Karanovic and Kim 2014b) and also for distinguishing closely related species using geometric morphometrics (Karanovic et al. 2016, 2018). However, in cyclopoids they seem to be more numerous, variable, and difficult to homologise (Karanovic and Blaha 2019).

Aims of this study were to describe four new species from South Korea in fine detail, asses their affinities using morphological characters, provide a global list of valid *Cyclopina* species and subspecies, and assemble a table of discrete and morphometric morphological characters most commonly used to identify species in this genus.

## Materials and methods

All specimens were collected from the intertidal zone in four localities in South Korea, using the Karaman-Chappuis method. This sampling technique involves digging a hole on the beach down to the water level and then decanting the inflowing interstitial water and filtering it through a plankton net (mesh size 30 μm). All samples were fixed in 99% ethanol, sorted in the laboratory also in 99% ethanol, using an Olympus SZX12 dissecting microscope with PLAPO objectives and magnification of up to 200 ×. Locality data and number of specimens are listed for each species separately and all material is deposited in the National Institute of Biological Resources (**NIBR**), Incheon, South Korea.

Some specimens were dissected and mounted on microscope slides in Faure’s medium (see Stock and von Vaupel Klein 1996), and dissected appendages were then covered by a coverslip. For the urosome, two human hairs of appropriate thickness were mounted between the slide and coverslip during examination, to prevent squashing. All line drawings were prepared using a drawing tube attached to a Leica MB2500 phase-interference compound microscope, equipped with N-PLAN (5 ×, 10 ×, 20 ×, 40 ×, and 63 × dry) or PL FLUOTAR (100 × oil) objectives. Specimens that were not drawn were examined in glycerol and, after examination, were stored in 99.9% ethanol. Specimens for scanning electron microscopy (SEM) were transferred into pure isoamyl-acetate for two hours, critical-point dried, mounted on stubs, coated in gold, and observed under a Hitachi S-4700 scanning microscope on the in-lens detector, with an accelerating voltage of 10 kV and working distances between 12 mm and 13.5 mm; micrographs were taken with a digital camera.

The terminology for morphological characters mostly follows Huys and Boxshall (1991), except for the numbering of setae on the caudal rami (not used) and small differences in the spelling of some appendages (antennula, mandibula, maxillula instead of antennule, mandible, maxillule); the latter as an attempt to standardise the terminology for homologous appendages in different crustacean groups. However, the terminology of maxilla and maxilliped follows revisions proposed by Ferrari and Ivanenko (2008). In order to save space and avoid unnecessary repetitions, species descriptions are comparative.

## Results

### Cyclopina busanensis sp. nov.

Taxon classificationAnimaliaCyclopoidaCyclopinidae

2A22F8DF-4A1F-5307-B8CB-E0C479A7FD77

http://zoobank.org/F8FFA47A-D9C4-4826-A6F1-5F286041EE1A

**Figure 1.**
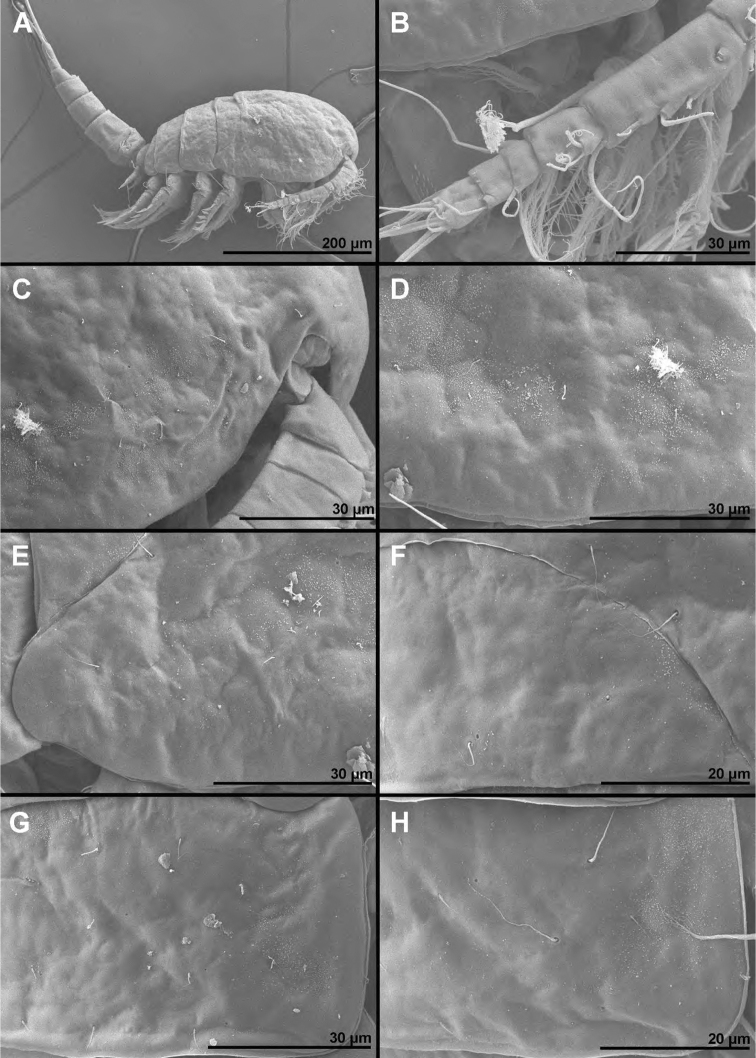
*Cyclopina busanensis* sp. nov., paratype female 1, SEM photographs, all in lateral view **A** habitus **B** distal part of antennula **C** anterior part of cephalothorax with rostrum **D** central part of cephalothoracic shield **E** postero-lateral corner of cephalothoracic shield **F** tergite of first pedigerous somite (= first free prosomite), mostly covered by postero-lateral corner of cephalothoracic shield **G** tergite of second pedigerous somite **H** tergite of third pedigerous somite.

**Figure 2.**
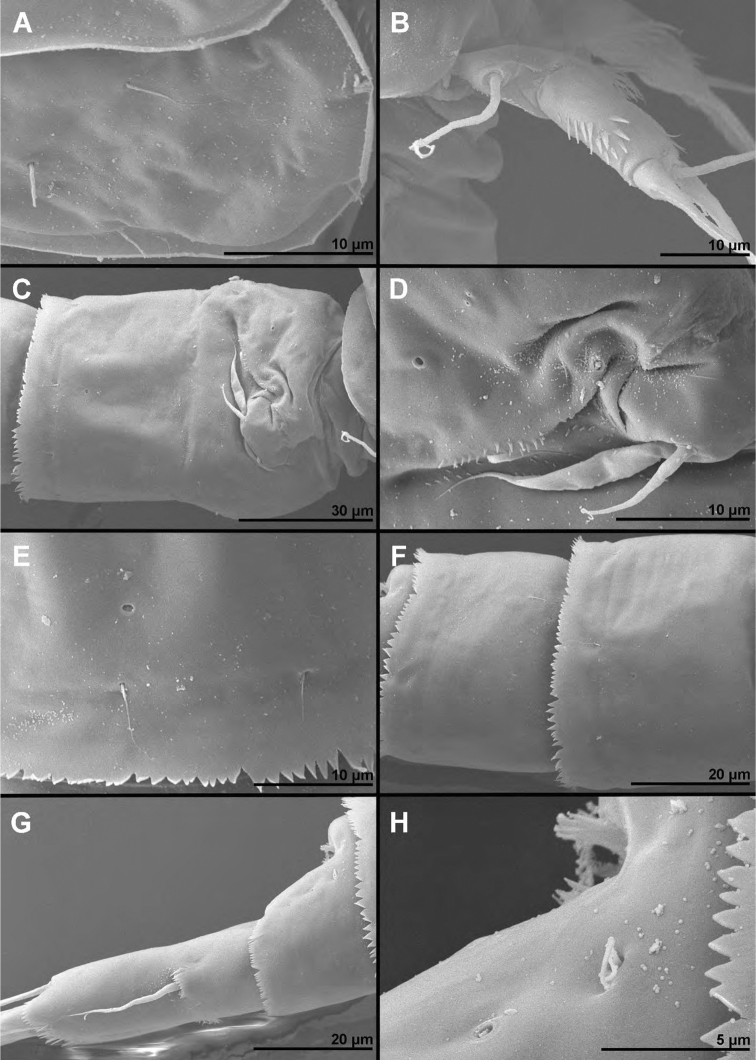
*Cyclopina busanensis* sp. nov., paratype female 1, SEM photographs, all in dorsal view **A** tergite of fourth pedigerous somite **B** fifth leg **C** genital double-somite (=fused second and third urosomites) **D** sixth leg **E** detail of posterior part of genital double-somite **F** fourth and fifth urosomites **G** sixth urosomite (= anal somite) and caudal ramus **H** detail of sixth urosomite.

**Figure 3.**
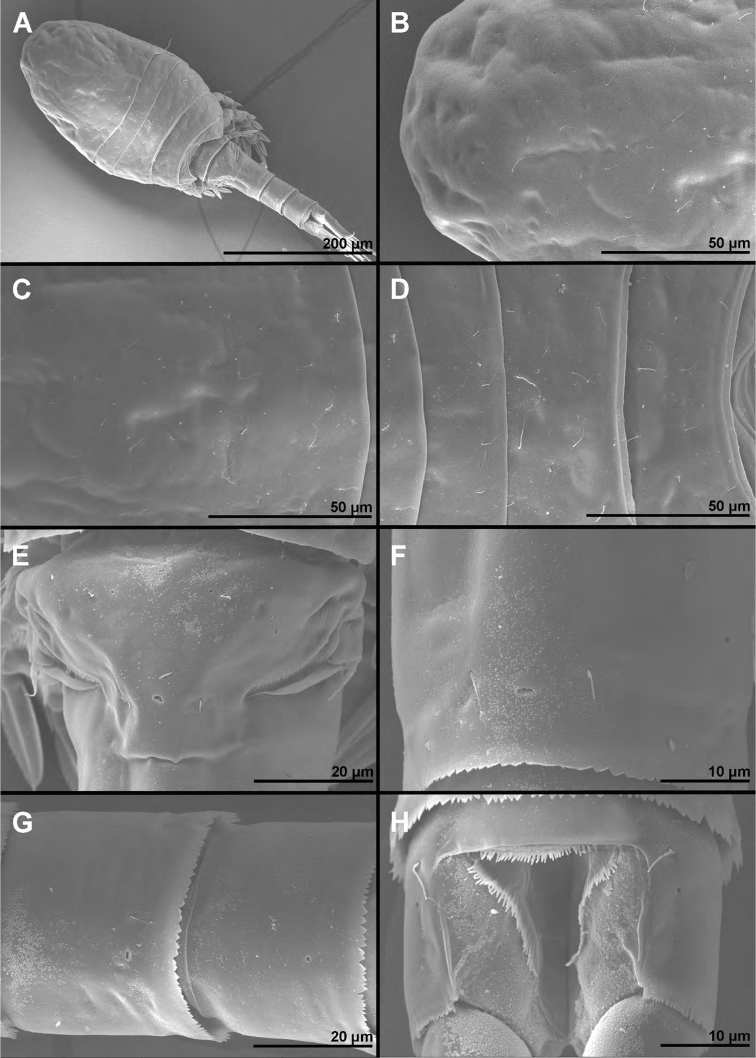
*Cyclopina busanensis* sp. nov., paratype female 2, SEM photographs, all in dorsal view **A** habitus **B** anterior part of cephalothorax **C** posterior part of cephalothorax **D** free prosomites **E** anterior part of genital double-somite **F** posterior part of genital double-somite **G** fourth and fifth urosomite **H** sixth urosomite.

**Figure 4.**
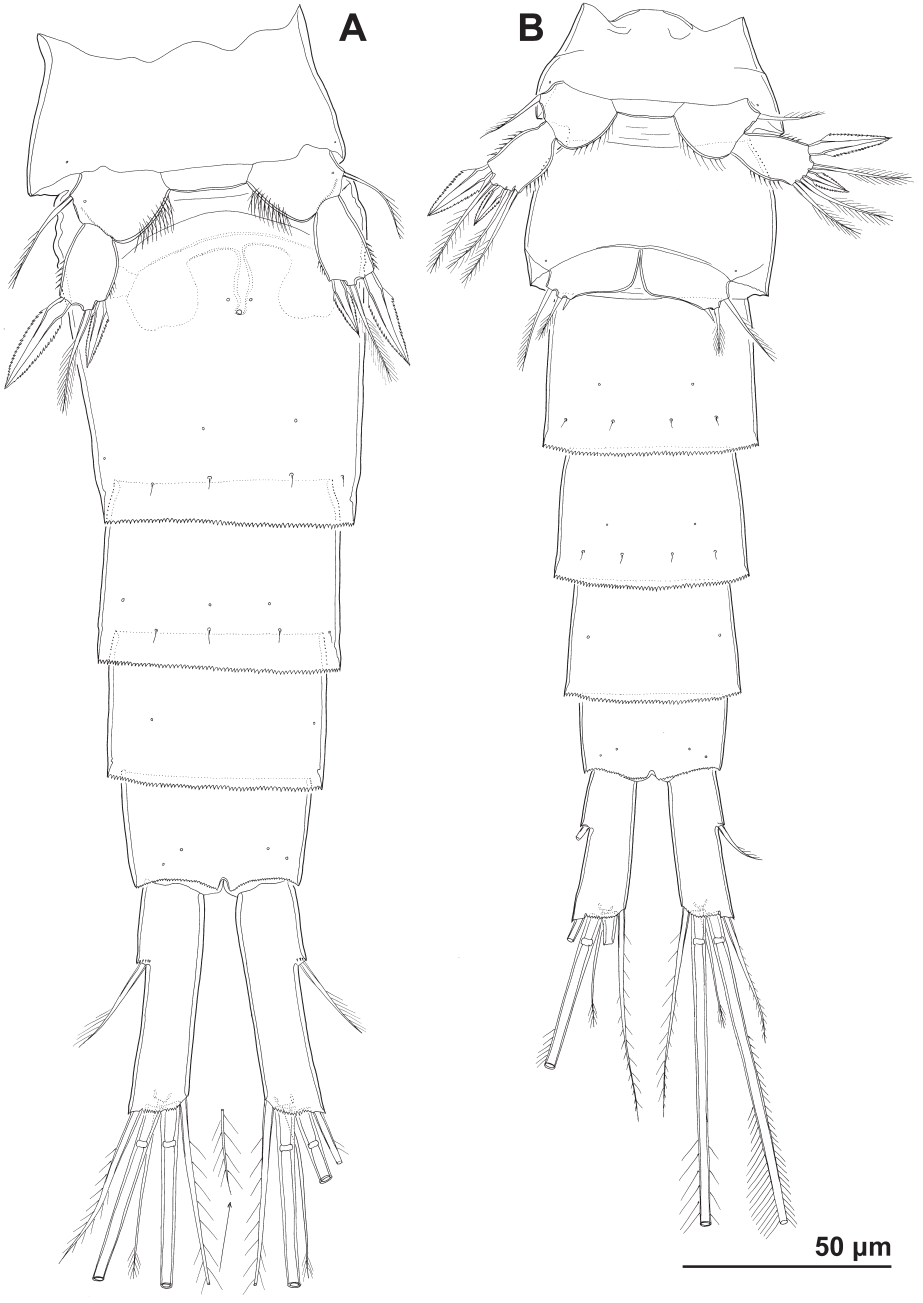
*Cyclopina busanensis* sp. nov., line drawings **A** holotype female, urosome, ventral view **B** allotype male, urosome, ventral view.

**Figure 5.**
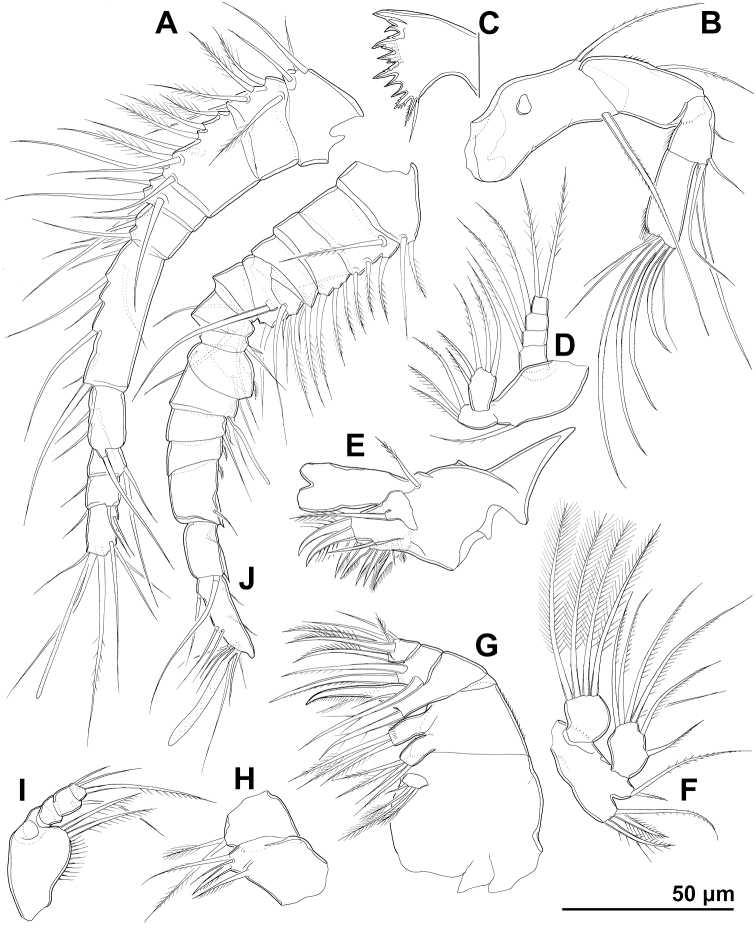
*Cyclopina busanensis* sp. nov., line drawings **A–I** holotype female **J** allotype male: **A** antennula **B** antenna **C** cutting edge of mandibula **D** mandibular palp **E** praecoxa of maxillula **F** maxillular palp **G** maxilla **H** syncoxa and basis of maxilliped **I** endopod of maxilliped **J** antennula.

**Figure 6.**
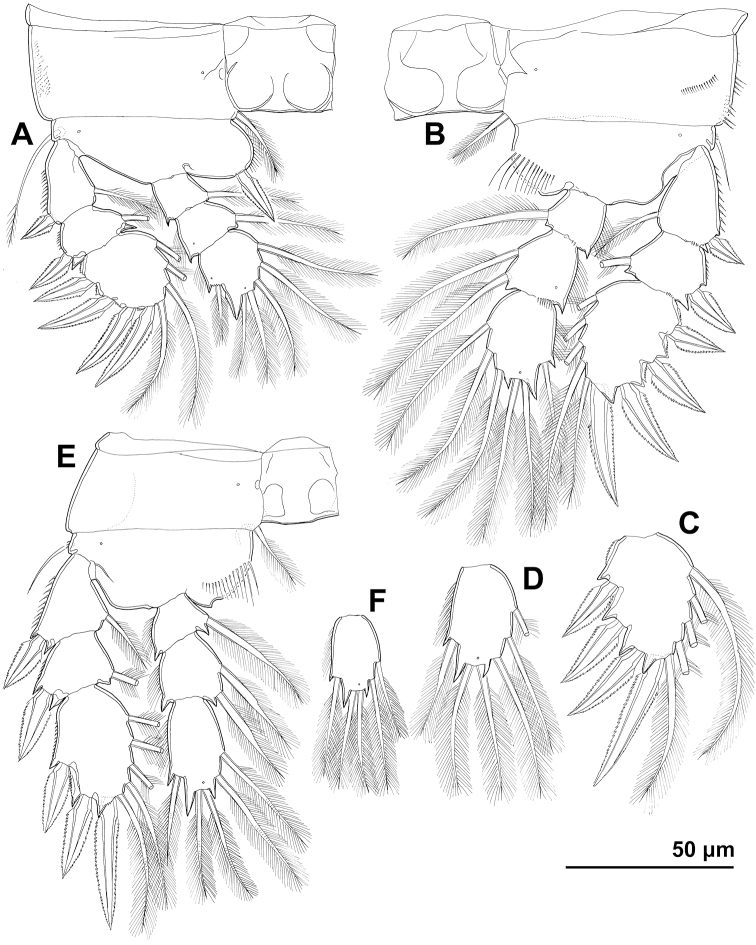
*Cyclopina busanensis* sp. nov., line drawings **A–E** holotype female **F** allotype male: **A** first swimming leg **B** second swimming leg **C** third exopodal segment of third swimming leg **D** third endopodal segment of third swimming leg **E** fourth swimming leg **F** third endopodal segment of fourth swimming leg.

Figures 1 , 2 , 3 , 4 , 5 , 6 , 17A

#### Type locality.

South Korea, South Coast, Busan, Sonjong Beach, intertidal sand, 35°10.741'N, 129°12.317'E.

#### Specimens examined.

***Holotype*** ovigerous female dissected on one slide, collected from the type locality, 6 May 2016, leg. T. Karanovic. ***Paratypes***: one male (allotype) and two females dissected on one slide each, seven females (one ovigerous) and five copepodids in alcohol, and five females on one SEM stub (together with specimens of other three species described here; row no. 2), all collected from the type locality, 6 May 2016, leg. T. Karanovic.

#### Etymology.

The species name refers to the type locality. It is an adjective for place, made with the Latin suffix -*ensis*.

#### Description.

**Female** (based on holotype and seven paratypes). ***Body length***, excluding caudal setae, from 515 to 535 μm. ***Colour*** of preserved specimens light brown, nauplius eye not visible (Fig. 17A). Integument on all somites smooth (Figs 1–3), with light bacterial cover, spinules only on genital and anal somites and caudal rami, cuticular pores on all somites, and sensilla on all but penultimate somite; hyaline fringes of prosomites smooth, of urosomites serrated. ***Habitus*** (Figs 1A, 3A) ca. 2.8 × as long as wide in dorsal view, with pronounced distinction between prosome and urosome; prosome ovoid, ca. 1.6 × as long as wide in dorsal view, nearly 1.3 × as long and 2.6 × as wide as urosome, its greatest width at posterior end of first pedigerous somite; urosome gently tapering towards posterior end, 3.3 × as long as wide, its greatest width at posterior end of fifth pedigerous somite (first urosomite). First pedigerous somite (Fig. 1A, F) not fused to cephalothorax, but its tergites partly covered with posterior extensions of cephalothoracic shield (Fig. 1A, E). Pedigerous somites without lateral expansions. ***Rostrum*** (Fig. 1C, B) well-developed, membranous, very broad. ***Cephalothorax*** (Figs 1A, C–E, 3B, C) nearly conical,approximately as long as wide, and 1.3 × as long as free prosomites combined. Second to fourth free prosomites (Figs 1A, G, H, 2A, 3D) progressively shorter and narrower towards posterior end, and with fewer cuticular organs.

***First urosomite*** (Figs 1A, 2C, 3A) shortest, laterally expanded in posterior part.

***Genital double-somite*** (Figs 2C–E, 3E, F, 4A) ca. 1.2 × as long as wide in dorsal view, laterally expanded anterior part nearly 1.4 × as wide as posterior margin; anterior part (second urosomite) with one pair of narrowly spaced posterior dorsal sensilla (Fig. 3E), large dorsal medial pore in between them (Fig. 3E), one pair of widely spaced anterior dorsal sensilla, one pair of small widely spaced anterior dorsal pores, one pair of narrowly spaced ventral pores next to copulatory pore (Fig. 4A), and two pairs of large pores, two pairs of small pores, and longitudinal row of spinules next to genital apertures (Fig. 2D); posterior part (third urosomite) with also with one pair of narrowly spaced posterior sensilla and large dorsal medial pore in between them (Fig. 3F), one pair of lateral posterior sensilla (Fig. 2E), one pair of large lateral pores (Fig. 2E), one pair of widely spaced ventral pores (Fig. 4A), and two pairs of posterior ventral sensilla (Fig. 4A). Medial copulatory pore (Fig. 4A) hardly bigger than cuticular pores next to it, situated in first third. Copulatory duct (Fig. 4A) narrow, rigidly sclerotised, T-shaped. Seminal receptacles (Fig. 4A) weakly sclerotised, simple, ovoid, with space between them slightly wider than one receptacle, reaching posteriorly slightly beyond level of copulatory pore. Oviducts weakly sclerotised, short. Genital apertures situated laterally, covered by reduced sixth legs. Paired egg sacs ovoid, each containing 8–10 eggs, twice as long and ca. 1.2 × as wide as genital double-somite. Fourth urosomite (Figs 2F, 3G, 4A) ca. 0.6 × as long as genital double-somite, with sensilla and pores as in third urosomite, except ventral pores situated slightly more posteriorly and more narrowly spaced. Fifth urosomite (Figs 2F, 3G, 4A) 0.8 × as long as fourth urosomite, with medial dorsal pore and one pair of widely spaced ventral pores. Sixth (anal) urosomite (Figs 2G, H, 3H, 4A) nearly 0.8 × as long as fifth urosomite, with one pair of large dorsal sensilla, one pair of dorsal pores, two pairs of ventral pores, and three rows of slender spinules fringing anal sinus; anal operculum smooth, short, broad, slightly concave, situated in first third, represents 66% of somite’s width.

***Caudal rami*** (Figs 2G, 4A) cylindrical, ca. 3.7 × as long as wide and twice as long as anal somite, narrowly spaced on anal somite, diverging posteriorly; armed with one proximal lateral seta, one dorsal seta, and four terminal setae; ornamented with row of small spinules at base of proximal lateral seta, and posterior ventral row of spinules. All setae slender and pinnate, and all except dorsal seta uni-articulated at base; two central terminal setae much longer and stronger than others and both with breaking planes; dorsal seta inserted close to posterior margin, biarticulated at base; proximal lateral seta inserted atapproximately two fifths of ramus’ length; medial terminal seta 1.2 × as long as caudal ramus, 1.6 × as long as lateral terminal seta, 1.5 × as long as dorsal seta, and 2.5 × as long as proximal lateral seta.

***Antennula*** (Figs 1A, B, 5A) reaching two thirds of cephalothoracic shield with its distal tip, stout, smooth, cylindrical but tapering towards distal end, 10-segmented; no setae with breaking planes or biarticulated, one seta on fifth segment short and spiniform, largest seta on ultimate segment and seven setae on second and third segments bipinnate, all other setae smooth and slender; single slender aesthetasc on ultimate segment fused basally to slender seta; armature formula (ae = aesthetasc) 3.5.8.4.5.7.4.3.2.7+ae; sixth segment longest, ca. 2.8 × as long as wide, and more than 0.8 × as long as subsequent four segments combined; tenth segment 1.5 × as long as wide.

***Antenna*** (Fig. 5B) slender, cylindrical, four-segmented, with highly mobile joint between second and third segment; first segment (probably allobasis) longest and widest, twice as long as wide, slightly curved, unornamented, armed with single strong medial-distal seta and twice as long exopodal seta; second segment (probably first endopodal) 0.8 × as long as basis, twice as long as wide, with spinules along medial convex margin, and with single medial seta inserted mid-length; second endopodal segment slightly narrower and only half as long as first endopodal, with spinules along lateral margin, and with four medial setae (shortest one inserted in proximal half, three near distal-medial corner; one distal seta spiniform, others slender); third endopodal segment 1.4 × as long as second endopodal and twice as long as wide, with spinules along lateral margin and seven apical setae (four strong and prehensile, three slender).

***Mandibula*** (Fig. 5C, D) with large coxa, and smaller palp consisting of basis, two-segmented endopod, and four-segmented exopod; coxal gnathobase with relatively wide cutting edge consisting of four polycuspidate large teeth (ventralmost largest), three smaller unicuspid teeth (dorsalmost with serrated edges, others smooth), row of spinules at base of two central polycuspidate teeth, and two short setae; dorsalmost seta on cutting edge smooth, ca. 1.5 × as long as other, bipinnate seta; basis ovoid, 1.7 × as long as wide, with single medial seta; endopod 0.6 × as long as basis, with three setae on first and five setae on second segment; exopod slightly shorter than basis but much more slender, with armature formula 1.1.1.2; all setae on basis, endopod, and exopod slender and pinnate.

***Maxillula*** (Fig. 5E, F) unornamented, composed of well-developed praecoxa and three-segmented palp; arthrite of praecoxa with six strong and pinnate apical spines, one isolated smooth spine on posterior surface, two spiniform plumose setae, and two smooth minute setae (or perhaps large spinules?) in between plumose setae and spines; proximalmost seta longest and strongest element, three × as long as other seta and ca. 1.1 × as long as longest and strongest (ventralmost) spine; coxa reduced to small endite partly fused to arthrite of praecoxa, bearing single slender seta, and another slender seta probably belonging to former epipodite; palp slightly smaller than praecoxa, composed of large rectangular basis, small ovoid endopod, and also ovoid but shorter and wider exopod; basis twice as long as wide, with short proximal and distal endites bearing three and two setae respectively; endopod slightly longer than greatest width of basis, ca. 1.5 × as long as wide, with two medial and four distal slender setae (one smooth, others unipinnate); exopod 0.8 × as long as endopod, as long as wide, with four distal slender and plumose setae.

***Maxilla*** (Fig. 5G) stout, 1.6 × as long as wide, tapering towards distal end, ornamented with row of spinules along lateral margin and several spinules on endites, composed of syncoxa (fused praecoxa and coxa), basis, and three-segmented endopod; syncoxa largest, quadrate, with four setae on proximal endite and one seta on distal endite; basis ca. 0.6 × as long as syncoxa, also quadrate, with three setae on proximal endite and three setae on distal endite; first endopodal segment half as long as coxa, with basally fused, smooth and robust claw and two articulated setae, proximal seta strong and bipinnate, slightly longer than claw, distal seta smooth and minute; second and third endopodal segments combined slightly longer than first, second segment somewhat longer than third and armed with four strong setae, third segment armed with three strong and three slender setae.

***Maxilliped*** (Fig. 5H, I) prehensile, slender, almost 3.5 × as long as wide, seven-segmented, composed of syncoxa, basis, and five-segmented endopod; syncoxa rhomboidal, approximately as long as wide, unornamented, with one element on proximal endite and three on distal endite; basis slightly smaller than syncoxa, quadrate, unornamented, with two setae on only endite; first endopodal segment nearly as long as syncoxa and basis combined, 1.6 × as long as wide, with row of long spinules along swollen medial margin, and with two spiniform setae near distal medial corner; distal part of endopod cylindrical, 0.7 × as long as basis, 2.4 × as long as wide, with armature formula 0.0.1.3, second endopodal segment partly fused to first endopodal and last segment half as long as any other; medial apical seta spiniform, 1.7 × as long as last four endopodal segments, twice as long as central apical seta, and 1.4 × as long as setae on first endopodal segment; other three endopodal setae slender.

***Swimming legs*** (Figs 1A, 6A–E) large, composed of short praecoxa, rectangular large coxa, triangular basis, three-segmented exopod, three-segmented endopod, and coxae of opposite appendages connected with squarish intercoxal sclerite; coxae of all legs with pore on anterior surface, row of spinules along lateral margin, and slender seta on medial-distal corner; intercoxal sclerites unornamented, with nearly straight distal margin; basis with slender lateral seta, anterior pore, row of long spinules along convex medial margin, row of minute spinules at base of lateral seta, and strong medial spine on first leg and short spiniform process instead on other legs; all exopodal segments with short spinules along lateral margin, and all endopodal segments with long and slender spinules along lateral margin; second endopodal segment of first to third leg with single anterior pore, third endopodal segment of first leg with two anterior pores, and third endopodal segments of second to fourth leg with single anterior pore; first and second exopodal segments with single lateral spine and single medial seta; first endopodal segments of all legs and second endopodal segment of first leg with single medial seta; second endopodal segments of second to fourth legs with two medial setae; third endopodal segments seta formula 6.6.6.5; third exopodal segment seta formula 4.5.5.5 and spine formula 4.4.4.3; third endopodal segment of fourth leg 1.7 × as long as wide and third exopodal segment of fourth leg ca. 1.5 × as long as wide; all setae slender and all spines lanceolate.

***Fifth leg*** (Figs 2B, 4A) small, two-segmented, with short intercoxal sclerite; first segment (presumably basis)approximately as long as wide, with single lateral seta, single anterior pore, several parallel rows of long spinules along convex medial margin, and distal row of minute spinules; second segment (presumably exopod) ca. 1.3 × as long as first but much narrower, 1.6 × as long as wide, with spinules along both medial and lateral slightly convex margins, apical central seta and two subapical spines; lateral spine 1.2 × as long as exopod and 1.6 × as long as medial spine.

***Sixth leg*** (Fig. 2D) simple semi-circular flap, mostly fused to genital somite, approximately twice as wide as long, unornamented, with two dorsally directed setae; lateral seta much stronger and nearly twice as long as medial seta.

**Male** (based on allotype). ***Body*** length 503 μm. ***Urosome*** (Fig. 4B) slenderer than in female, and second and third urosomites fully articulated; ornamentation as in female, except ventral pores on third and fourth urosomites more widely spaced.

***Caudal rami*** (Fig. 4B) slightly shorter than in female, but armature and ornamentation without significant differences.

***Antennula*** (Fig. 5J) digeniculate, 15-segmented, with proximal geniculation between eighth and ninth and segments, and distal geniculation between thirteenth and fourteenth segments; armature formula: 2.5.4.2.6.1.1.2.2.1+ae.2.1.2.1.11+ae; thirteenth and fourteenth segments with strong cuticular ridges along anterior (geniculating) surface; ninth, eleventh, twelfth, and thirteenth segments with short spiniform seta each, all other setae slender and most also smooth.

***Antenna, mandibula, maxillula, maxilla, maxilliped***, and all four swimming legs as in female. Third endopodal segment of fourth leg (Fig. 6F) ca. 1.5 × as long as wide.

***Fifth leg*** (Fig. 4B) segmentation, ornamentation, and armature of proximal segment as in female; armature of distal segment with two slender medial setae in addition to two spines and central apical seta as in female; lateral spine as long as distal segment and ca. 1.8 × as long as medial spine.

***Sixth leg*** (Fig. 4B) also simple semi-circular flap, but better articulated than in female, with medial minute spine and two slender setae; lateral seta 1.7 × as long as central seta and more than 5 × as long as spine.

#### Variability.

Cuticular organs on the cephalothorax (Figs 1C–E, 3B, C) often exhibited asymmetries in position and/or absence on one side and in different specimens, to the point that a complete survey was probably impossible. Cuticular organs on free prosomites showed fewer asymmetries in position (Fig. 3D) and rarely any absence, while those on urosomites showed no variability in position or number (Figs 2D, 3E). There was no variability in the segmentation or armature formulae of appendages, and any variability in the proportion of segments or armature elements could not be confidently discounted as resulting from slight difference in position due to mounting of specimens and appendages.

### Cyclopina koreana sp. nov.

Taxon classificationAnimaliaCyclopoidaCyclopinidae

F3BBB514-15CF-5E45-B107-6667E97AEF1B

http://zoobank.org/9ACBBC99-22DF-4150-8938-666F7099CC05

**Figure 7.**
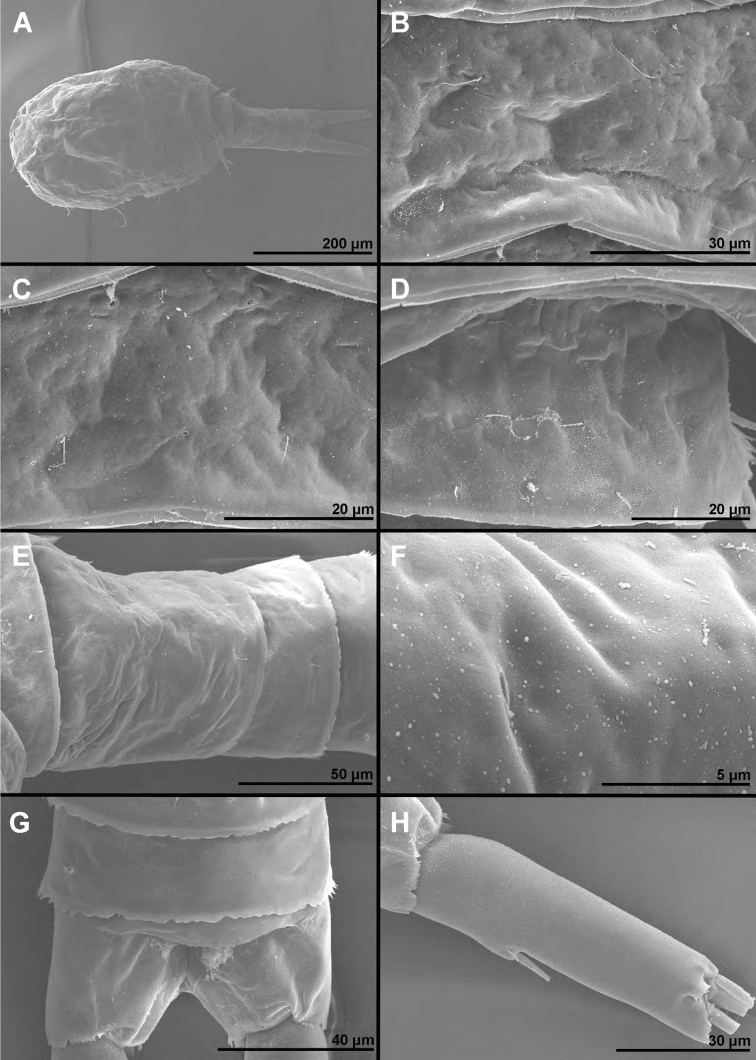
*Cyclopina koreana* sp. nov., paratype female 1, SEM photographs, all in dorsal view **A** habitus **B** second pedigerous somite **C** third pedigerous somite **D** first urosomite **E** genital double-somite and fourth urosomite **F** anterior medial pore on genital double-somite **G** fifth and sixth urosomites **H** caudal ramus.

**Figure 8.**
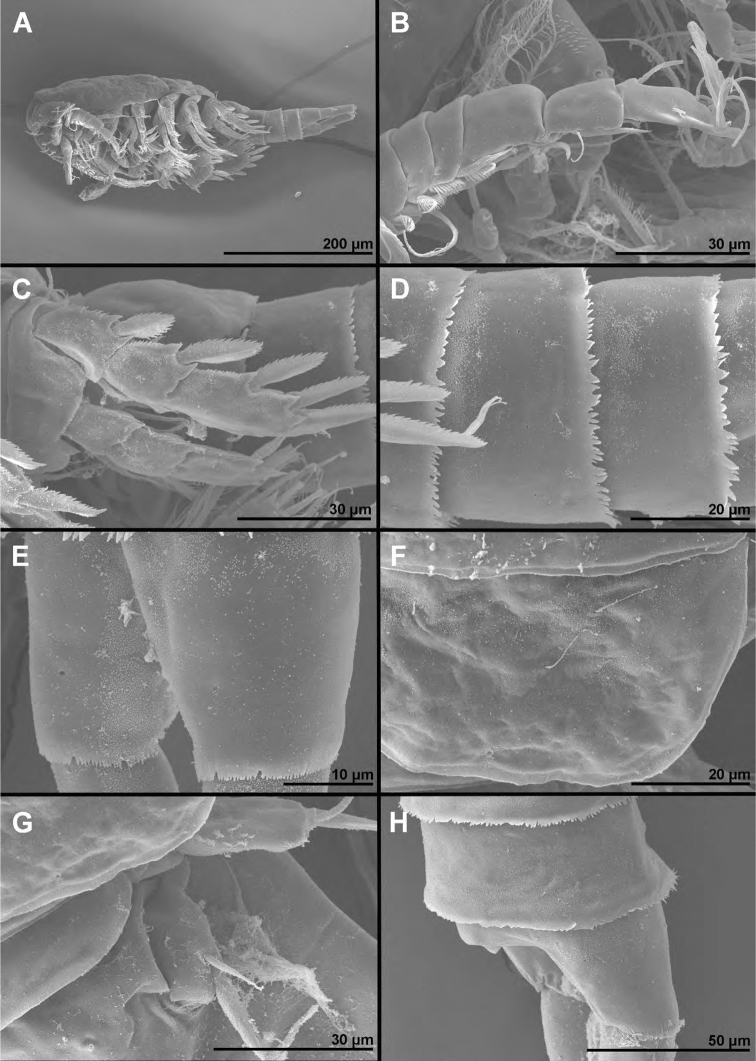
*Cyclopina koreana* sp. nov., SEM photographs **A–E** paratype male 1, ventral view **F–H** paratype female 2, lateral view: **A** habitus **B** distal part of antennula **C** fourth swimming leg **D** fourth and fifth urosomites **E** sixth urosomite **F** tergite of fourth pedigerous somite **G** anterior part of urosomite, with fifth and sixth legs **H** fifth and sixth urosomites.

**Figure 9.**
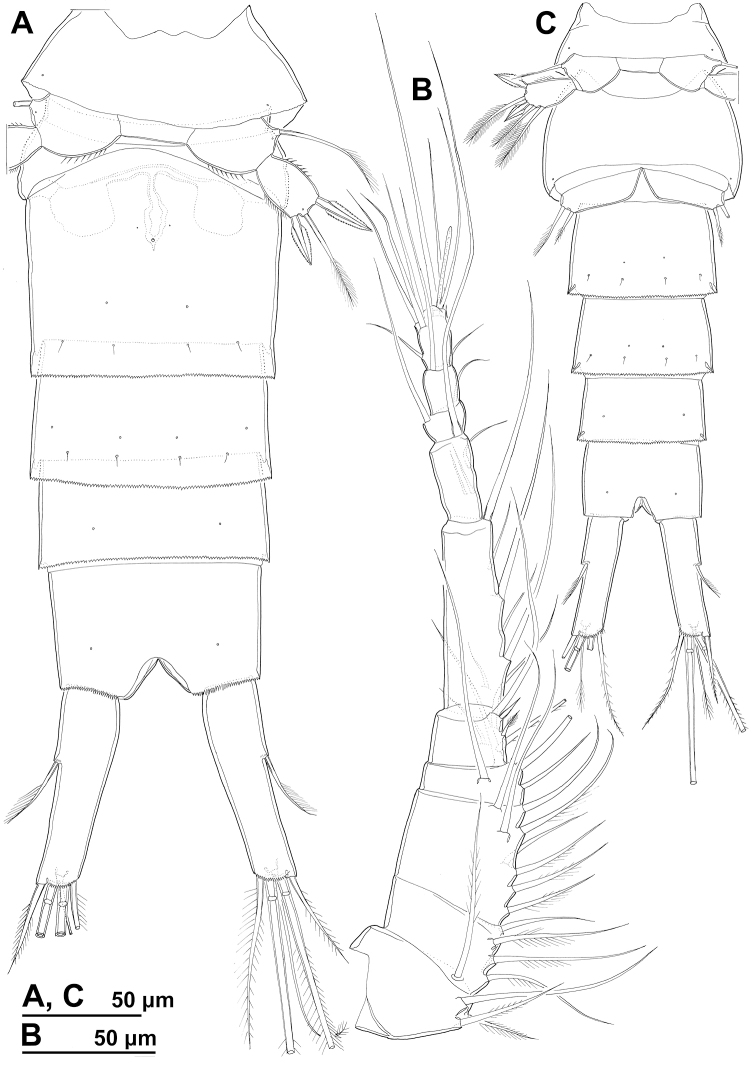
*Cyclopina koreana* sp. nov., line drawings **A** holotype female, urosome, ventral view **B** holotype female, antennula **C** allotype male, urosome, ventral view.

**Figure 10.**
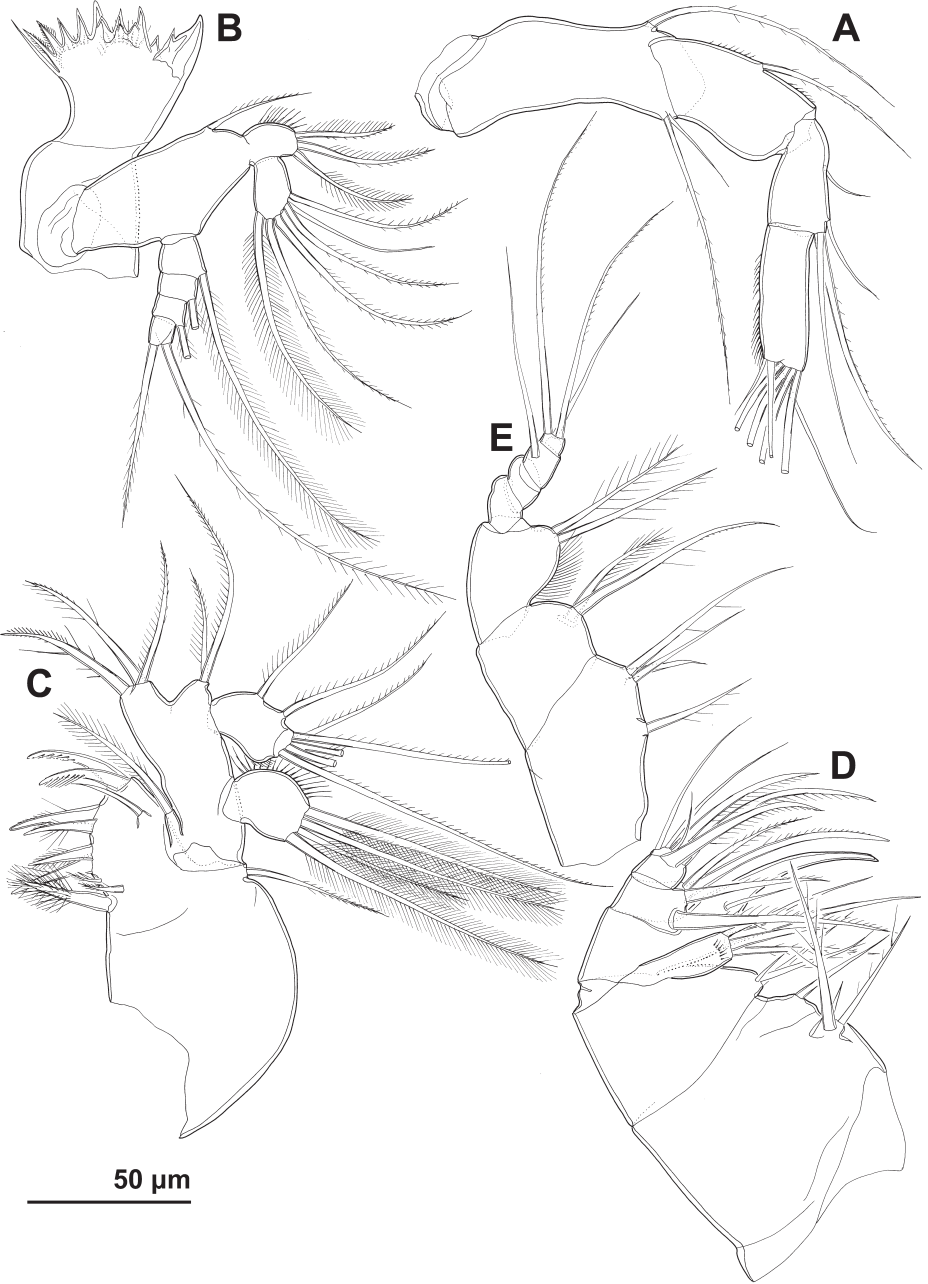
*Cyclopina koreana* sp. nov., line drawings, holotype female **A** antenna **B** mandibula **C** maxillula **D** maxilla **E** maxilliped.

**Figure 11.**
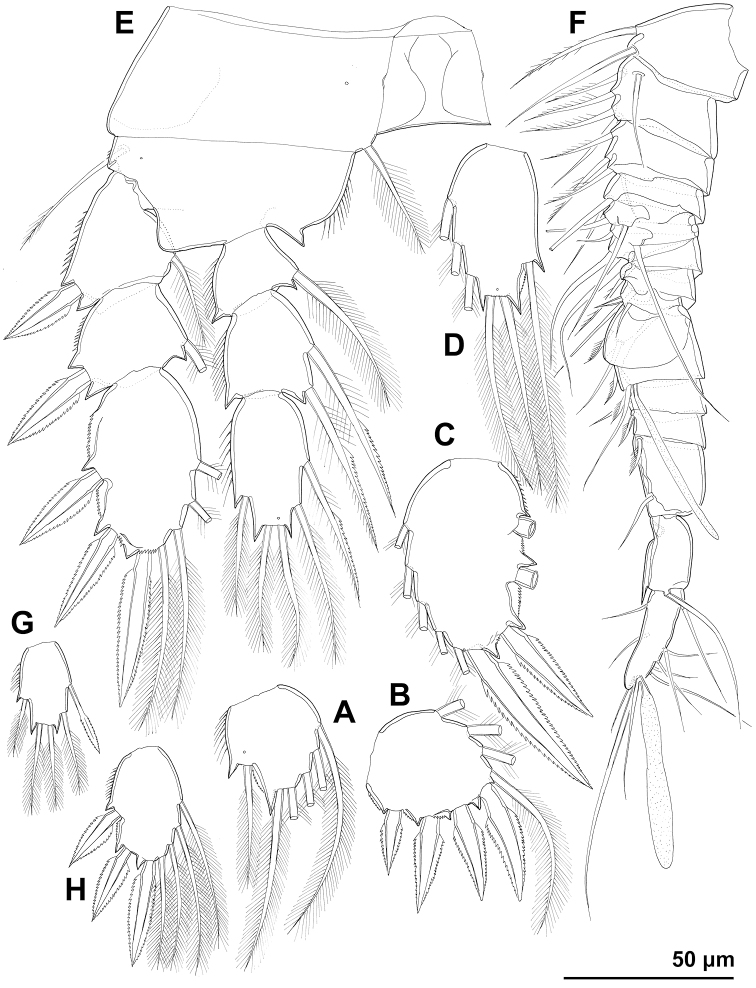
*Cyclopina koreana* sp. nov., line drawings **A–E** holotype female **F–H** allotype male: **A** third endopodal segment of first swimming leg **B** third exopodal segment of first swimming leg **C** third exopodal segment of second swimming leg **D** third endopodal segment of second swimming leg **E** fourth swimming leg **F** antennula **G** third endopodal segment of fourth swimming leg **H** third exopodal segment of fourth swimming leg.

Figures 7 , 8 , 9 , 10 , 11 , 17B

#### Type locality.

South Korea, East Coast, Gangneung, small beach, intertidal sand, 37°47.824'N, 128°55.085'E.

#### Specimens examined.

***Holotype*** female dissected on one slide, collected from the type locality, 29 March 2013, leg. T. Karanovic.

***Paratypes***: two males and one female dissected on one slide each; three males, two females, and four copepodids in alcohol; one male and two females on one SEM stub (together with specimens of other three species described here; row no. 4); all collected from the type locality, 29 March 2013, leg. T. Karanovic.

#### Etymology.

The species name refers to South Korea. It is an adjective, agreeing in gender with the feminine genus name.

#### Description.

**Female** (based on holotype and three paratypes). ***Body length*** from 620 to 635 μm. ***Colour*** of preserved specimens yellowish, nauplius eye not visible (Fig. 17B). Integument on all somites (Figs 7, 8) smooth, with light bacterial cover, cuticular pores on all somites, spinules only on genital somite and caudal rami, and sensilla on all but penultimate somite; hyaline fringes of prosomites smooth, of urosomites serrated. ***Habitus*** (Fig. 7A) ca. 2.6 × as long as wide in dorsal view, with pronounced distinction between prosome and urosome; prosome ovoid, ca. 1.5 × as long as wide in dorsal view, nearly 1.4 × as long and 2.6 × as wide as urosome, its greatest width at posterior end of first pedigerous somite; urosome nearly cylindrical, ca. 3 × as long as wide, its greatest width at posterior end of fifth pedigerous somite (first urosomite). ***First pedigerous somite*** (Fig. 7A) not fused to cephalothorax, but its tergites partly covered with posterior extensions of cephalothoracic shield as in *C. busanensis*. ***Cephalothorax*** (Fig. 7A) broader in anterior part than in *C. busanensis*, ca. 1.2 × as long as wide, and twice as long as free prosomites combined. Second to fourth free prosomites (Figs 7A–C, 8F) progressively shorter and narrower towards posterior end, and with fewer cuticular organs; not many prosomal cuticular organs clearly homologous to those in previous species (compare Figs 2A, 8F), except dorsal medial pores and several posterior sensilla (Fig. 7B, C).

***First urosomite*** (Figs 7D, 8G, 9A) short, slightly laterally expanded in posterior part, with four dorsal sensilla and single dorsal medial pore.

***Genital double-somite*** (Figs 7E, F, 8G, 9A) ca. 0.9 × as long as wide in dorsal view, laterally expanded anterior part only ca. 1.1 × as wide as posterior margin; sensilla and pores as in *C. busanensis*. Copulatory pore, copulatory duct, seminal receptacles, oviducts, and genital apertures as in *C. busanensis*, except first part of copulatory duct slightly wider. Fourth urosomite (Figs 7E, 9A) ca. 0.6 × as long as genital double-somite, with sensilla and pores as in *C. busanensis*. Fifth urosomite (Figs 7G, 8H, 9A) 0.7 × as long as fourth urosomite, with medial dorsal pore and one pair of widely spaced ventral pores as in *C. busanensis*. Sixth urosomite (Figs 7G, 8H, 9A) 1.2 × as long as fifth urosomite, with one pair of dorsal sensilla, two pairs of dorsal pores, and single pair of ventral pores; no spinules on fringes of anal sinus; anal operculum smooth, short, broad, slightly convex, situated in first third, represents 62% of somite’s width.

***Caudal rami*** (Figs 7H, 8H, 9A) cylindrical, ca. 3.5 × as long as wide and 1.5 × as long as anal somite, very widely spaced on anal somite, diverging posteriorly; armed as in *C. busanensis*; ornamented with single sensilla near proximal lateral seta, row of small spinules at base of proximal lateral seta, and posterior ventral row of spinules. Proximal lateral seta inserted at ca. two fifths of ramus’ length; medial terminal seta nearly 0.9 × as long as caudal ramus, 1.6 × as long as lateral terminal seta, 0.8 × as long as dorsal seta, and 2.3 × as long as proximal lateral seta.

***Antennula*** (Fig. 9B) segmentation and most armature as in *C. busanensis*, but proximal half stouter and distal half slenderer; armature formula 3.6.8.4.5.6.4.2.2.7+ae; apical aesthetasc significantly shorter than in *C. busanensis* and fifth segment with two short setae; sixth segment longest, ca. 3 × as long as wide, and nearly 0.9 × as long as subsequent four segments combined; tenth segment nearly twice as long as wide.

***Antenna*** (Fig. 10A) as in *C. busanensis*, but another small exopodal seta present and second endopodal segment slightly longer.

***Mandibula*** (Fig. 10B) as in *C. busanensis*, except second endopodal segment with six setae, apical setae on fourth exopodal segment of markedly different lengths (outer one twice as long as inner one), and additional row of minute spinules at base of unicuspid teeth.

***Maxillula*** (Fig. 10C) segmentation and armature formula as in *C. busanensis*, but only one minute seta on praecoxal arthrite smooth, one seta on endopod markedly shorter than other endopodal setae, and both endopod and exopod slightly slenderer.

***Maxilla*** (Fig. 10D) as in *C. busanensis*, but with only three setae on proximal syncoxal endite, proximal basal endite less mobile and with one seta minute, endopodal claw smooth, and endopod four-segment.

***Maxilliped*** (Fig. 10E) segmentation and armature formula as in *C. busanensis*, but with longer syncoxa, shorter first endopodal segment, and two long setae on ultimate endopodal segment.

***Swimming legs*** (Fig. 10A–E) shape, segmentation, and ornamentation as in *C. busanensis*; armature formula as in *C. busanensis*, except third exopodal segment of fourth leg with only four setae; all spines lanceolate; three setae on endopod of fourth leg also lanceolate, other setae slender; third endopodal segments seta formula 6.6.6.5; third exopodal segment seta formula 4.5.5.4 and spine formula 4.4.4.3; third endopodal segment of fourth leg 1.7 × as long as wide and third exopodal segment of fourth leg ca. 1.6 × as long as wide.

***Fifth leg*** (Fig. 8A, G) shape, segmentation, armature formula, and ornamentation as in *C. busanensis*, but first segment slightly shorter (ca. 0.6 × as long as wide) and lateral spine on second segment also proportionately shorter (approximately as long as second segment and 1.3 × as long as medial spine).

***Sixth leg*** (Fig. 8G) as in *C. busanensis*.

*Male* (based on allotype and two other paratypes). ***Body*** length from 440 to 500 μm. Habitus (Fig. 8A) similar to female, but slenderer. ***Urosome*** (Figs 8D, E, 9C) also slenderer than in female, and second and third urosomites fully articulated as in *C. busanensis*; ornamentation as in female.

***Caudal rami*** (Fig. 9C) slightly less widely spaced than in female, but armature and ornamentation without significant differences (perhaps dorsal seta somewhat shorter).

***Antennula*** (Figs 8B, 11F) shape, geniculation, segmentation, ornamentation, and almost all armature as in *C. busanensis*, but fifth segment with traces of additional segmentation, both aesthetascs longer, and tenth segment with additional short seta (armature formula therefore: 2.5.4.2.6.1.1.2.2.2+ae.2.1.2.1.11+ae).

***Antenna***, mandibula, maxillula, maxilla, maxilliped, and all four swimming legs (Fig. 8A, C) as in female. Third endopodal segment of fourth leg (Fig. 11G) ca. 1.6 × as long as wide, with proximal medial seta lanceolate along both sides; third exopodal segment (Fig. 11H) with only four setae as in female, ca. 1.6 × as long as wide.

***Fifth leg*** (Fig. 9C) segmentation, ornamentation, and armature formula as in *C. busanensis*, i.e., with two medial setae on second segment; proximal segment as in female; lateral spine ca. 0.7 × as long as second segment and 1.1 × as long as medial spine.

***Sixth leg*** (Fig. 9C) as in *C. busanensis*, but broader and without minute medial spine; lateral seta 1.5 × as long as medial seta.

#### Variability.

Except for small differences in body size no other forms of variability were observed, but some specimens were damaged (e.g., with some setae broken off; see Fig. 7H) so comparisons were somewhat limited.

### Cyclopina curtijeju sp. nov.

Taxon classificationAnimaliaCyclopoidaCyclopinidae

577C84E8-9712-568F-8C39-0DA201132A5A

http://zoobank.org/D1D520C8-2BC3-4A0B-B00C-A9BBC730D4A8

**Figure 12.**
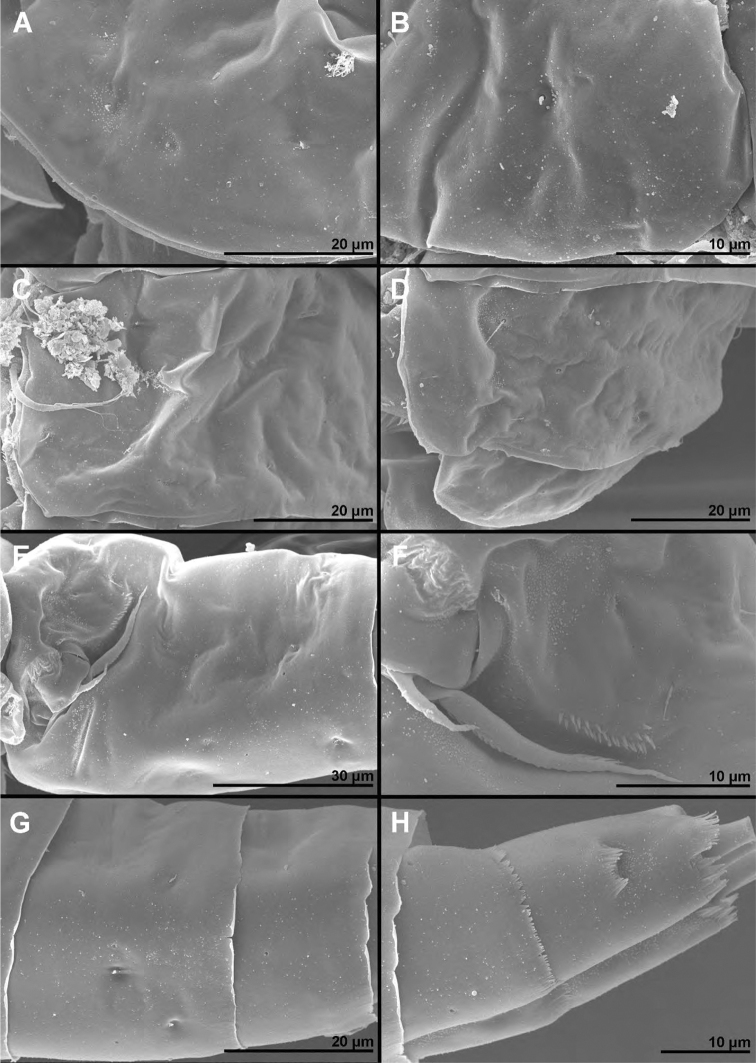
*Cyclopina curtijeju* sp. nov., paratype female, SEM photographs, all in lateral view **A** anterior part of cephalothoracic shield **B** posterio-lateral corner of cephalothoracic shield **C** tergite of second pedigerous somite **D** tergites of third and fourth pedigerous somites **E** genital double-somite **F** sixth leg **G** fourth and fifth urosomites **H** sixth urosomite and caudal rami.

Figures 11 , 12 , 17C

#### Type locality.

South Korea, South Coast, Jeju Island, Gwangchigi Beach near Seongsan Sunrise Peak, 33°27.122'N, 126°55.481'E.

#### Specimens examined.

***Holotype*** female dissected on one slide, collected from the type locality, 14 April 2014, leg. T. Karanovic. ***Paratype*** female on an SEM stub (together with specimens of other three species described here; row no. 3), collected from the type locality, 14 April 2014, leg. T. Karanovic.

#### Etymology.

The species name is composed of the Latin adjective *curtus* (= short), referring to its short caudal rami, and the name of the type locality (Jeju). It should be treated as a noun (gender feminine) in apposition to the generic name.

#### Description.

**Female** (based on holotype and one paratype). ***Body length*** 400 μm. ***Colour*** (Fig. 17C), nauplius eye, body segmentation, integument on somites (Fig. 12), and general habitus as in *C. busanensis*, except for different sensilla and pores pattern on prosomites (Fig. 12A–D) and hyaline fringes of urosomites (except anal somite) rather wavy than serrated (Fig. 12F–H). Prosome ca. 1.4 × as long as urosome.

***Genital double-somite*** (Fig. 12E, F) as in *C. busanensis*, except with two additional dorsolateral pair of pores, one additional ventrolateral pair of pores, posterior lateral pore almost at same level as posterior lateral sensillum (instead of being anterior to posterior lateral sensillum), and sensillum instead of large pore at dorsal end of lateral row of spinules. Genital field not clearly observed because of mounting, but in lateral view seems very similar to that in *C. busanensis*. Fourth urosomite (Fig. 12G) as in *C. busanensis*, except with one additional pair of dorsal pores in anterior half. Fifth urosomite (Fig. 12G) and sixth urosomite (Fig. 12H) as in *C. busanensis*.

***Caudal rami*** (Fig. 12A, H) cylindrical, ca. 1.3 × as long as wide, 1.2 × as long as anal somite, narrowly spaced on anal somite, parallel; ornamented as in *C. busanensis*; most armature broken off; dorsal seta nearly 3 × as long as ramus; proximal lateral seta inserted at approximately midlength of ramus.

**Figure 13.**
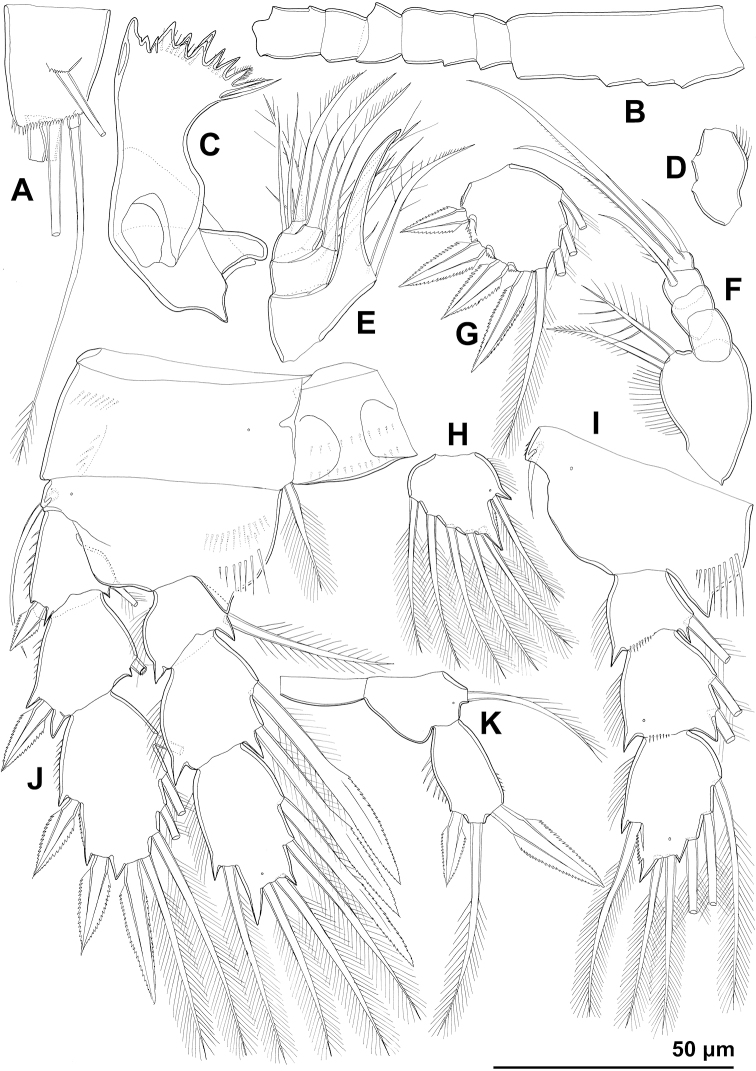
*Cyclopina curtijeju* sp. nov., holotype female, line drawings **A** caudal ramus, lateral view **B** distal part of antennula, without armature **C** coxa of mandibula **D** endopod of maxillula **E** endopod of maxilla **F** endopod of maxilliped **G** third exopodal segment of first swimming leg **H** third endopodal segment of first swimming leg **I** basis and endopod of second swimming leg **J** fourth swimming leg **K** fifth leg.

***Antennula*** (Fig. 13B) 11-segmented, but all armature as in *C. koreana*; armature formula 3.6.8.4.5.6.2.2.2.2.7+ae; sixth segment ca. 2.7 × as long as wide, nearly 0.9 × as long as subsequent five segments combined; tenth segment 1.5 × as long as wide.

***Antenna and mandibula*** (Fig. 13C) shape, segmentation, armature, and ornamentation as in *C. koreana*.

***Maxillula*** (Fig. 13D) also as in *C. koreana*, except endopod slightly slenderer.

***Maxilla*** (Fig. 13E) with only two setae on second endopodal segment; everything else as in *C. koreana*.

***Maxilliped*** (Fig. 13F) generally as in *C. koreana*, except first endopodal segment slightly slenderer, seta on fourth endopodal segment shorter, large setae on fifth endopodal segment stronger, and slender seta on fifth endopodal segment shorter.

***Swimming legs*** (Fig. 13G–J) shape, segmentation, armature formula, and most ornamentation as in *C. busanensis*; fourth leg (Fig. 13J) with three setae on endopod lanceolate as in *C. koreana*, five setae on third exopodal segment as in *C. busanensis*, but unlike these species with two parallel posterior rows of spinules on intercoxal sclerite and with posterior row of spinules on basis; third endopodal segment of fourth leg 1.6 × as long as wide and third exopodal segment of fourth leg ca. 1.5 × as long as wide.

***Fifth leg*** (Fig. 13K) shape, segmentation, and armature formula as in *C. busanensis*, but first segment without inner spinules and second segment slightly longer (1.5 × as long as first segment and 1.9 × as long as wide); lateral spine ca. 1.3 × as long as second segment and nearly 1.8 × as long as medial spine.

***Sixth leg*** (Fig. 12F) as in *C. busanensis*.

**Male** unknown.

#### Variability.

Only two females were examined, both partly damaged, one in detail with a light microscope (holotype), and the other with a scanning electron microscope (paratype), so variability could not be properly assessed. However, the paratype female was also beforehand examined with a light microscope (although without dissection) and no variability was observed in the most important diagnostic characters (caudal rami length, antennula segmentation, swimming legs armature, or fifth leg proportions); mouth appendages could not be examined without dissection.

### Cyclopina wido sp. nov.

Taxon classificationAnimaliaCyclopoidaCyclopinidae

5E8508BF-2F67-5D7C-9948-AE60917326A8

http://zoobank.org/06FD35E8-BD0D-4592-BAE3-717CEB70AF9C

**Figure 14.**
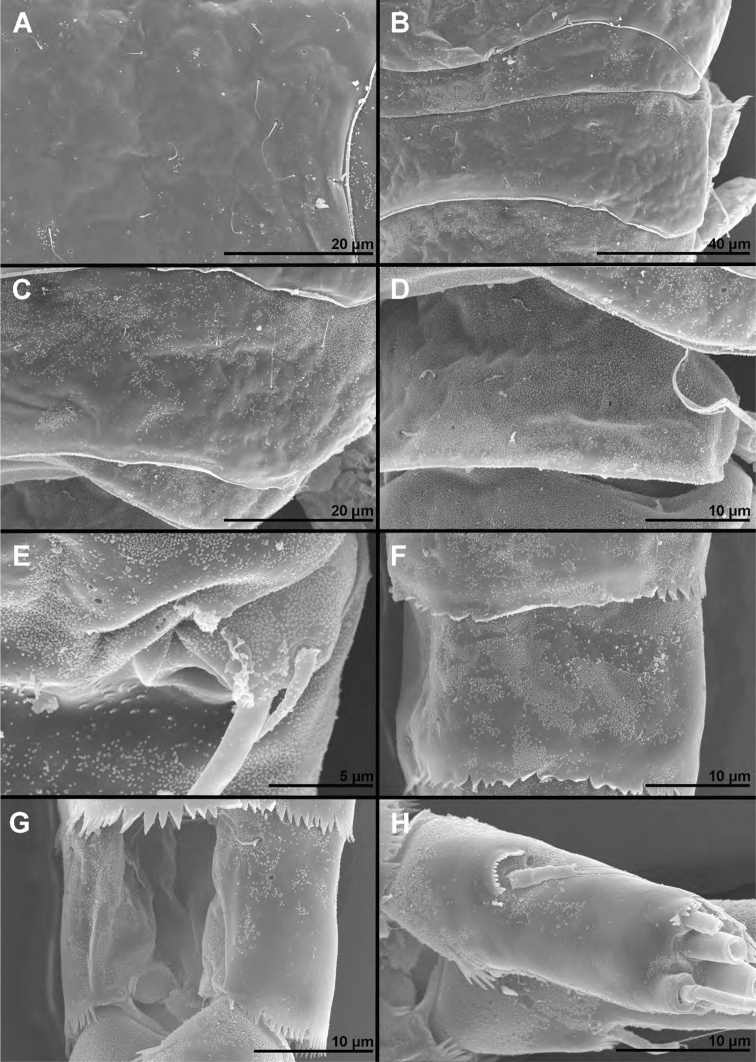
*Cyclopina wido* sp. nov., paratype female 1, SEM photographs, all in dorsal view **A** posterior part of cephalothorax **B** first and second pedigerous somites **C** third and fourth pedigerous somites **D** first urosomite **E** sixth leg **F** posterior part of genital double-somite and fourth urosomite **G** hyaline fringe of fifth urosomite and sixth urosomite **H** caudal rami.

**Figure 15.**
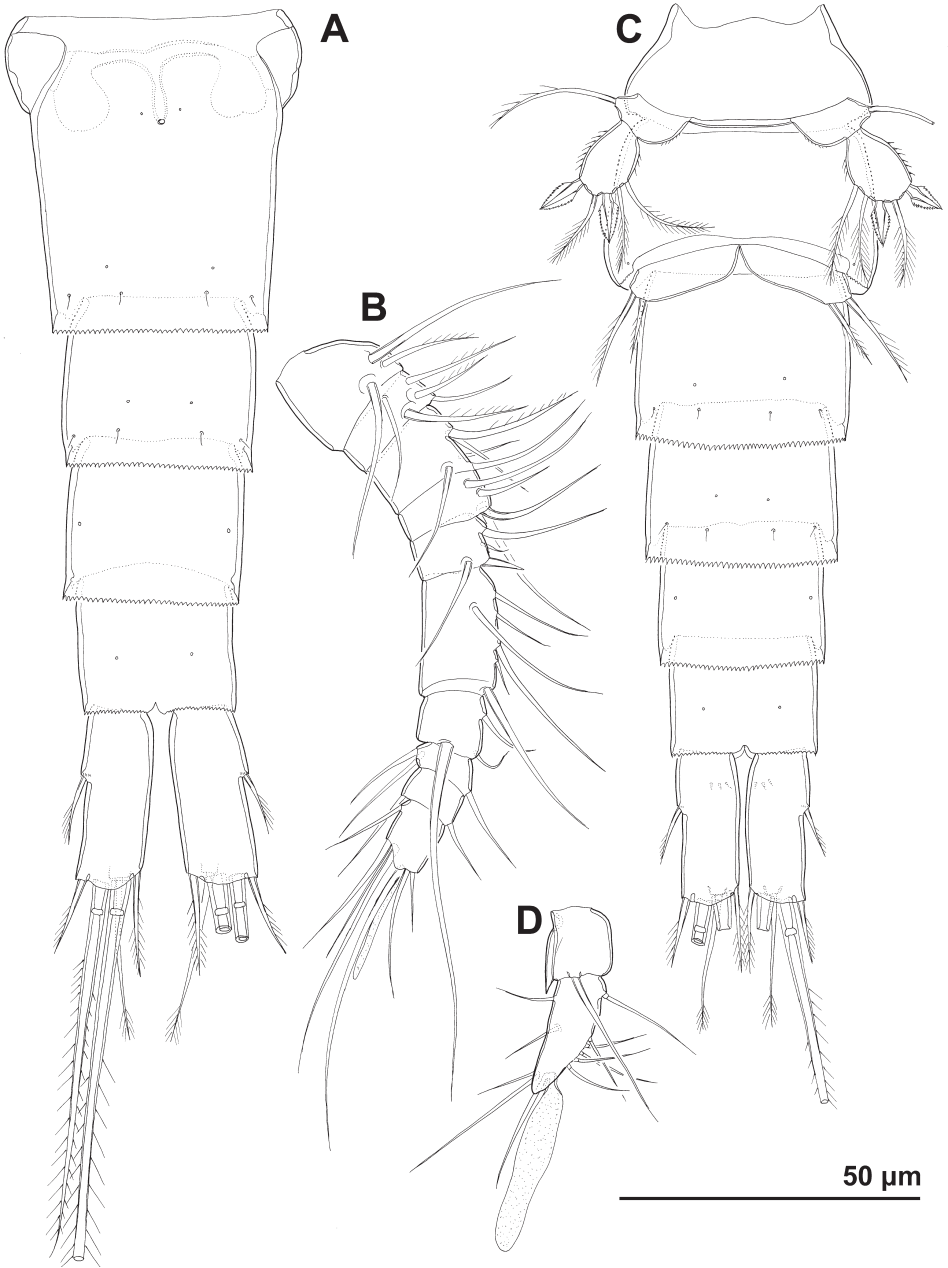
*Cyclopina wido* sp. nov., line drawings **A, B** holotype female **C, D** allotype male: **A** urosome without first urosomal somite, ventral view **B** antennula **C** urosome, ventral view **D** penultimate and ultimate segments of antennula.

**Figure 16.**
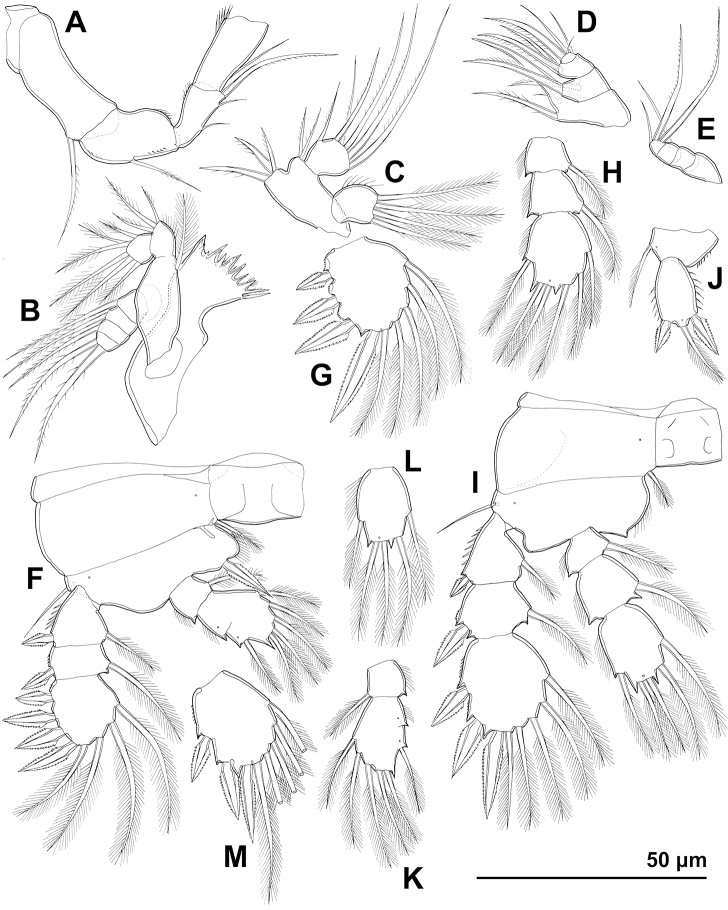
*Cyclopina wido* sp. nov., line drawings **A–J** holotype female **K–M** allotype male: **A** antenna, without apical armature **B** mandibula **C** maxillular palp **D** endopod of maxilla **E** last four endopodal segments of maxilliped **F** first swimming leg **G** third exopodal segment of second swimming leg **H** endopod of second swimming leg **I** fourth swimming leg **J** fifth leg **K** endopod of first swimming leg **L** third endopodal segment of fourth swimming leg **M** third exopodal segment of fourth swimming leg.

**Figure 17.**
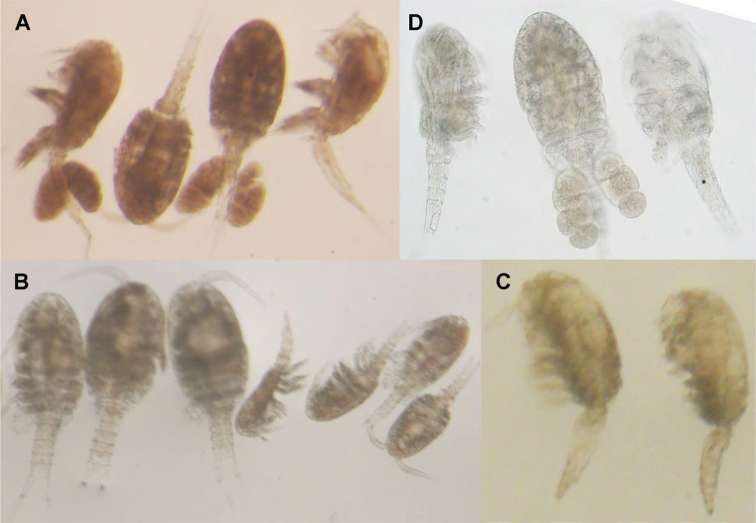
Light photographs of four new species (not to scale) **A***Cyclopina busanensis* sp. nov., four females (two ovigerous) **B***Cyclopina koreana* sp. nov., three females and four males **C***Cyclopina curtijeju* sp. nov., two females **D***Cyclopina wido* sp. nov., two females (one ovigerous) and one male.

Figures 14 , 15 , 16 , 17D

#### Type locality.

South Korea, West Coast, Wido Island, small beach, intertidal sand, 35°35.089'N, 126°15.196'E.

#### Specimens examined.

***Holotype*** ovigerous female dissected on one slide, collected from the type locality, 12 April 2013, leg. T. Karanovic.

***Paratypes***: one male (allotype) dissected on one slide; one female on one SEM stub (together with specimens of other three species described here; row no. 1); both collected from the type locality, 12 April 2013, leg. T. Karanovic.

#### Etymology.

The species name refers to its type locality (Wido). It should be treated as a noun (gender feminine) in apposition to the generic name.

#### Description.

**Female** (based on holotype and one paratype). ***Body length*** of holotype 327 μm, that of paratype 323 μm. ***Colour*** of preserved specimens yellowish, nauplius eye not visible (Fig. 17D). Integument on all somites (Fig. 14) smooth, with moderate bacterial cover, cuticular pores on all somites, spinules only on genital somite and caudal rami, and sensilla on all but penultimate somite; hyaline fringes of prosomites smooth, of urosomites serrated. ***Habitus*** ca. 2.6 × as long as wide in dorsal view, with pronounced distinction between prosome and urosome; prosome ovoid but with more flared posterior end than in *C. busanensis*, ca. 1.6 × as long as wide in dorsal view, nearly 1.6 × as long and 2.7 × as wide as urosome, its greatest width at posterior end of first pedigerous somite; urosome nearly cylindrical, ca. 3 × as long as wide, its greatest width at posterior end of fifth pedigerous somite (first urosomite). ***First pedigerous somite*** (Fig. 14B) not fused to cephalothorax, but its tergites largely covered with posterior extensions of cephalothoracic shield. ***Cephalothorax*** (Fig. 14A) shape as in *C. busanensis*, nearly conical, approximately as long as wide, and 1.2 × as long as free prosomites combined; however, cuticular sensilla and pores pattern unique. Second to fourth free prosomites (Fig. 14B, C) progressively shorter and narrower towards posterior end, and with fewer cuticular organs; not many prosomal cuticular organs obviously homologous to those in previous species, except perhaps dorsal medial pores and several posterior sensilla.

***First urosomite*** (Fig. 14D) as in *C. busanensis* and *C. koreana*, short, slightly laterally expanded in posterior part, with two pairs of dorsal sensilla, single dorsal medial pore, one pair of dorsolateral pores, and one pair of ventrolateral pores (at base of fifth legs).

***Genital double-somite*** (Figs 14E, 15A) as in *C. busanensis*, except ventral posterior pores slightly closer to ventral posterior sensilla, pair of small lateral anterior pores closer to sixth leg, somite ca. 1.1 × as long as wide, and laterally expanded anterior part nearly 1.4 × as wide as posterior margin. Copulatory pore, copulatory duct, seminal receptacles, oviducts, and genital apertures as in *C. busanensis*. Fourth urosomite (Figs 14F, 15A) ca. half as long as genital double-somite, with sensilla and pores as in *C. busanensis*. Fifth urosomite (Figs 14G, 15A) almost as long as fourth urosomite, with medial dorsal pore and one pair of widely spaced ventral pores as in *C. busanensis*, but dorsal hyaline fringe coarsely serrated and expanded posteriorly almost as pseudo-operculum (completely covering anal operculum). Sixth urosomite (Figs 14G, 15A) 0.85 × as long as fifth urosomite, with one pair of dorsal sensilla, two pairs of dorsal pores, and single pair of ventral pores; no spinules on fringes of narrow anal sinus; anal operculum smooth, very short, narrow, slightly concave, situated in first fourth, represents approximately 40% of somite’s width.

***Caudal rami*** (Figs 14H, 7H, 8H, 9A) robust, cylindrical, ca. 2.3 × as long as wide and 1.4 × as long as anal somite, very narrowly spaced on anal somite, nearly parallel; armed with six setae as in *C. busanensis*; ornamented with single pore near proximal lateral seta, row of small spinules at base of proximal lateral seta, posterior ventral row of spinules, and short diagonal dorsomedial row of large spinules in anterior half. Proximal lateral seta inserted at ca. two fifths of ramus’ length; medial terminal seta only ca. 0.6 × as long as caudal ramus, 1.2 × as long as lateral terminal seta, 0.6 × as long as dorsal seta, and 1.6 × as long as proximal lateral seta.

***Antennula*** (Fig. 15B) 10-segmented, very stout, nearly cylindrical (proximal part only slightly wider than distal part); armature formula 3.5.5.4.4.6.3.3.2.7+ae; apical aesthetasc significantly shorter than in *C. busanensis* and fifth segment with two short setae, as in *C. koreana*; sixth segment longest, ca. 1.5 × as long as wide, and 0.6 × as long as subsequent four segments combined; tenth segment ca. 1.3 × as long as wide.

***Antenna*** (Fig. 16A) shape, segmentation, most ornamentation, and most armature as in *C. koreana*, but no exopodal setae and inner-distal seta on basis significantly shorter.

***Mandibula*** (Fig. 16B) as in *C. koreana*, except cutting edge somewhat narrower, basis slenderer, exopod stouter, setae on fourth exopodal segment of equal length, and no spinules at base of unicuspid teeth.

***Maxillula*** (Fig. 16C) as in *C. koreana*, except endopod shorter and with only six setae, as well as setae on distal basal endite of equal length.

***Maxilla*** (Fig. 16D) as in *C. curtijeju*, i.e., with only two setae on second endopodal segment.

***Maxilliped*** (Fig. 16E) as in *C. koreana*, except apical setae slightly shorter.

***Swimming legs*** (Fig. 16F–I) shape, most segmentation, most ornamentation, and most armature as in *C. busanensis*, except endopod of first leg two-segmented and with one less seta, as well as second endopodal segments of second to fourth legs with single medial seta; all spines lanceolate and all setae slender; third exopodal segment seta formula 4.5.5.5 and spine formula 4.4.4.3; third endopodal segment of fourth leg 1.3 × as long as wide and third exopodal segment of fourth leg only ca. 1.1 × as long as wide.

***Fifth leg*** (Fig. 16J) shape, segmentation, armature formula, and ornamentation as in *C. koreana*, but second segment longer and lateral spine shorter than medial; second segment ca. 1.9 × as long as first segment and ca. 1.7 × as long as wide; lateral spine ca. 0.5 × as long as second segment and 0.7 × as long as medial spine.

***Sixth leg*** (Fig. 14E) as in *C. busanensis*.

**Male** (based on allotype). ***Body length*** 305 μm. ***Habitus*** similar to female, but slightly slenderer. ***Urosome*** (Fig. 15C) also slenderer than in female, and second and third urosomites fully articulated as in *C. busanensis*; ornamentation as in female.

***Caudal rami*** (Fig. 15C) slightly shorter and slenderer than in female, but armature and ornamentation without significant differences.

***Antennula*** (Fig. 15D) geniculation, segmentation, ornamentation, and all armature as in *C. koreana*, but all segments shorter.

***Antenna, mandibula, maxillula, maxilla, maxilliped***, and all four swimming legs (Fig. 16K–M) as in female. Endopod of first leg (Fig. 16K) also two-segmented, with only seven setae; third endopodal segment of fourth leg (Fig. 16L) nearly 1.4 × as long as wide; third exopodal segment of fourth leg (Fig. 16M) ca. 1.2 × as long as wide.

***Fifth leg*** (Fig. 15C) segmentation, ornamentation, and armature formula as in *C. busanensis*, except second segment more rounded and lateral spine shorter; second segment twice as long as first segment and 1.5 × as long as wide; lateral spine ca. 0.6 × as long as distal segment and 0.9 × as long as medial spine.

***Sixth leg*** (Fig. 15C) without medial spine as in *C. koreana*; lateral seta 1.4 × as long as medial seta.

#### Variability.

Only one male and two females were examined, so variability could not be properly assessed. One female was examined in detail with a light microscope (holotype), and the other with a scanning electron microscope (paratype). However, the paratype female was also beforehand examined with a light microscope (although without dissection) and no variability was observed in the most important diagnostic characters (caudal rami length, antennula segmentation, swimming legs segmentation and armature, or fifth leg proportions); mouth appendages could not be examined without dissection. Male characters that are not sexually dimorphic show only minute differences from female characters in proportions of somites, segments, or armature.

## Discussion

Four new species from South Korea share a number of characters that are considered important in cyclopoid taxonomy and systematics, such as free first pedigerous prosomite, extended postero-lateral corners of the cephalothoracic shield, T-shaped copulatory duct and ovoid seminal receptacles on the completely fused genital double-somite, very short anal operculum, relatively short caudal rami armed with only six setae, short female antennula (10- or 11-segmented) with longest sixth segment, four-segmented antenna with armature formula of the last three segments 1/4/7, four-segmented mandibular exopod with armature formula 1/1/1/2, one-segmented maxillular exopod armed with four setae, maxillipedal armature formula 4/2/2/0/0/1/3, three-segmented exopods of all swimming legs with spine formula of the third segments 4/4/4/3, three-segmented endopods of second to fourth legs with seta formula of the third segments 6/6/5, two-segmented female fifth leg with two spines and central seta on the second segment, and male fifth legs (in three species with known males) with two additional medial setae on the second segment. All of these characters are within the currently recognised boundaries of the genus *Cyclopina* (see Lotufo 1994; Gómez and Martínez Arbizu 2004; Karanovic 2008). However, the four South Korean congeners can easily be distinguished from each other by a multitude of features, including size, caudal rami shape, proportions of caudal setae, proportions of ultimate endopodal and exopodal segments on the fourth leg, proportions of segments and armature on the fifth leg, and cuticular sensilla and pores pattern on prosomites and some urosomites. Other distinguishing characters include: space between caudal rami (less than the width of one ramus in *C. busanensis*, *C. curtijeju*, and *C. wido*; more than the width of one ramus in *C. koreana*) , antennula segmentation (10-segmented in *C. busanensis*, *C. koreana*, and *C. wido*; 11-segmented in *C. curtijeju*), number of exopodal setae on the antenna (one in *C. busanensis*, two in *C. koreana* and *C. curtijeju*, and none in *C. wido*), number of setae on the mandibular endopod (five in *C. busanensis*, six in other species), number of setae on the second endopodal segment of maxilla (four in *C. busanensis* and *C. koreana*, two in *C. curtijeju* and *C. wido*), number of long setae on the ultimate segment of maxilliped (one in *C. busanensis*, two in other species), first leg endopod segmentation (two-segmented in *C. wido*, three-segmented in other species), number of setae on the first leg endopod (seven in *C. wido*, eight in other species), number of setae on the second endopodal segment of second to fourth legs (one in *C. wido*, two in other species), number of setae on the third exopodal segment of fourth leg (four in *C. koreana*, five in other species), and nature of setae on the second endopodal segment of fourth leg (plumose in *C. busanensis* and *C. wido*, lanceolate in *C. koreana* and *C. curtijeju*). Other smaller differences are highlighted in their comparative descriptions above. It should be clear from the presented distribution of character states among the four species that there are no clear sister-species pairs here. The only thing about their phylogenetic relationships that can be concluded from morphological characters is that *C. wido* stands apart from the other three species by a number of reductions in segmentation and armature, which are perhaps related to its diminutive size.

Cuticular organs (sensilla and pores) on somites certainly show some differences between the four new South Korean species described here, but some of these rarely studied micro-characters could easily be homologised (especially on urosomites) and showed little intraspecific variability. This could be invaluable in future studies trying to match opposite sexes, especially because numerous *Cyclopina* species are known after only one sex (Karanovic 2008). It might also be useful in reconstructing difficult phylogenetic relationships among cyclopinids at large, as was shown for some harpacticoid copepods (Karanovic and Kim 2014b).

*Cyclopina busanensis* is probably most similar to the Japanese *C. kiraensis* Horomi, 1984, described from the Pacific Coast of Honshu (Hiromi 1984) and later reported from the same island, but from the Sea of Japan (Ueda et al. 2001). However, the Japanese species can easily be distinguished from its South Korean congener by slightly shorter caudal rami and ultimate endopodal and exopodal segments of the fourth leg, as well as by the presence of lanceolate setae on the fourth leg endopod and modified apical seta on the mandibular exopod. Both share many morphological details with a large group of species around the widely distributed *C. gracilis* Claus, 1863 (which is the type species of the genus) and the Mediterranean *C. esilis* Brian, 1928 (see Claus 1863; Sars 1913; Brian 1928; Steuer 1940; Lang 1946; Herbst 1964; Pallares 1968; Monchenko 1979; Wells and McKenzie 1973; Jaume and Boxshall 1996), but can be distinguished by at least some details in the proportion of certain segments and armature (Table 1). It should be noted that this whole complex is in need of revision, with intraspecific variability between some highly disjunct populations sometimes exceeding interspecific variability. For example, specimens redescribed as *C. esilis* from the Black Sea by Monchenko (1979) almost certainly represent a different species from those redescribed from Mallorca by Jaume and Boxshall (1996). Discrepancies in body size and caudal rami shape in some Mallorcan specimens reported by Jaume and Boxshall (1996) could indicate further sympatric cryptic species, as recently demonstrated using molecular tools for several groups of copepods (Karanovic and Cooper 2012; Karanovic et al. 2016). However, Jaume and Boxshall (1996) were probably well justified in synonymising with *C. esilis* specimens from France that were tentatively reported by Herbst (1953) as *C. kieferi* Schëfer, 1936. Problems surrounding distribution and variability of *C. gracilis* are of similar nature: while Sars (1913) stated that the male fifth leg is exactly the same as in the female in a population from Norway, Herbst (1964) illustrated a male fifth leg with one additional medial seta in a population from the Red Sea, and Pallares (1968) redescribed a population from Argentina that cannot possibly be conspecific with these two.

**Table 1.** List of selected character states for valid species and subspecies of the genus *Cyclopina* Claus, 1963. Abbreviations used: ?, unknown; +, present; –, absent; A, anterior; A1, antennula; A2, antenna; AnSo, anal somite; Bp, basis; Cr, caudal ramus; Enp, endopod; Exp, exopod; L, length; Md, mandibula; Mxl, maxillula; Mxp, maxilliped; P, posterior; P1, first leg; P4, fourth leg; P5, fifth leg; P6, sixth leg; W, width. See text for more details.

	Cr, L/W	Cr /AnSo	medial seta/Cr	Cr, medial/dist. lateral seta	Cr, medial/dorsal seta	Cr, prox. lat. seta position	Cr, space between rami	Female A1, segmentation	A2, no. of exopodal setae	A2Bp, no. of medial setae	A2Enp2, no. of setae	MdEnp, armature formula	MxlEnp, no. of setae	MxpEnp, armature formula	P1Enp, no. of segments	P1Enp, no. of setae	P4Exp3, L/W	P4Exp3, no of setae	P4Enp3, L/W	P4Enp, lanceolate setae	P5, Exp/Bp	P5Exp, L/W	P5, lateral spine/Exp	P5Exp, lateral/medial spine	Male P5, no. of medial setae	Male P6, medial spine
*C. adelphe* Karanovic, 2008	3	1.2	?	?	?	A	2.2	11	1	2	5	3/4	6	0/0/1/3	3	7	?	5	1.4	–	1.9	2.5	0.4	0.7	?	?
*C. adriatica* Petkovski, 1955	1.6	1	3	1.5	1.1	P	0.3	10	1	1	5	3/6	7	0/0/1/3	?	8	?	5	?	?	2.1	2.2	0.9	1	?	?
*C. americana* Herbst, 1982	1.5	1	2.7	2	1.5	P	0.5	10	0	1	4	3/6	?	0/0/1/2	3	?	1.5	5	1.5	–	1.5	2	1.3	2.5	1	–
*C. amita* Karanovic, 2008	1.8	0.9	2.3	2.2	1.2	P	0.4	11	1(0)	1(2)	4(5)	3/4	6	0/0/0/3	3	8	1.7	5	1.5	–	1.4	2.1	1.3	2.2	2	–
*C. arenosa* Lotufo, 1994	3.4	1.4	0.8	1.4	1.2	A	0.6	10	2	1	5	3/5	7	?	2	8	1.4	5	?	?	1.2	1.5	1	1.2	?	?
*C. balearica* (Jaume & Boxshall, 1996)	2.9	1.2	1.7	2.9	1.7	A	0.7	10	2	1	5	3/6	7	0/0/4	3	7	1.6	5	1.5	–	1.4	2.4	0.9	1.2	2	+
*C. brachystylis* Sars, 1921	1.5	0.9	2.7	1.5	1.4	P	0.2	10	?	?	?	?	?	?	?	?	?	?	?	?	1.2	2.2	1.3	2	?	?
*C. brevifurca* Sars, 1913	0.8	0.5	4.6	1.1	1	P	0.3	12	1	1	4	3/6	7	1/1/2	3	8	1.9	5	1.7	–	1.4	2.5	1.5	1.7	?	?
*C. busanensis* sp. nov.	3.7	2	1.2	1.6	1.5	A	0.5	10	1	1	4	3/5	7	0/0/1/3	3	8	1.5	5	1.6	–	1.3	1.6	1.2	1.6	2	+
*C. caiala* Lotufo & Rocha, 1991	1.7	0.8	2.6	1.7	2	P	0.2	10	1	1	5	3/6	7	1/0/1/3	3	8	1.3	5	1.3	+	1.5	1.6	0.5	1	0	+
*C. caissara* Lotufo, 1994	1.5	0.9	3.1	1.2	1.3	P	0.2	12	1	1	5	3/6	7	0/0/1/4	3	8	1.8	5	1.8	–	0.9	2	1.7	6.5	2	+
*C. campechana* (Suarez & Almeyda, 2015)	1.2	1	3	1.5	1.3	P	0.5	10	1	1	5	3/5	7	0/0/1/4	3	8	1.4	5	1.4	–	1.5	2	0.9	2.4	2	+
*C. caroli* Lotufo, 1994	1.5	1.1	3.5	2.2	2.3	P	0.3	10	2	1	5	3/5	7	0/0/1/4	3	8	1.5	4	1.4	+	1.7	1.7	1	1.3	1	+
*C. confusa* (Ivanenko & Defaye, 2004)	3.7	1.3	1.5	1.7	1.7	A	0.6	10	2	1	5	3/6	7	0/0/1/4	3	8	1.4	5	1.5	–	1.3	2.1	0.8	1.4	2	+
*C. crassisetosa* Herbst, 1953	3.7	1.4	1.1	1.9	0.7	P	0.3	10	0	1	4	3/?	7	0/0/1/3	3	?	?	?	1.4	–	1.6	1.5	1	1	?	?
*C. curtijeju* sp. nov.	1.3	1.2	?	?	?	P	0.3	11	2	1	4	3/6	7	0/0/1/3	3	8	1.5	5	1.6	+	1.5	1.9	1.3	1.8	?	?
*C. dorae* Lotufo, 1994	1.9	1.1	1.1	1.3	0.8	P	0.6	10	2	1	5	3/5	7	0/0/1/4	3	8	1.5	5	?	?	1.3	2	0.7	0.9	?	?
*C. ensifera* Grandori, 1925	3	1.4	1.7	1.6	1.4	P	0.3	10	1	1(0)	5	3/6	7	0/0/1/3	3	8	1.3	5	1.7	+	2	2	1.1	1.1	0	–
*C. esilis* Brian, 1938	3	1.5	1.6	2.5	1.5	A(P)	0.6	10	1(0)	1(0)	5	3/6	7	0/0/1/3(4)	3	8	1.8	5	2	+(–)	1.4	1.7	1.1	2.8	2	+(–)
*C. gracilis* Claus, 1863	4	2	0.8	1.2	0.8	A	0.8	10	1	1	4	2/6	6	0/1/2	3	8	1.8	5	1.4	–	1.2	1.3	1.5	1.2	1(0)	+
*C. hadzii* Petkovski, 1955	2.8	1.7	0.7	1.7	1	A	0.5	10	1	1	3	?	?	?	?	7	?	5	?	?	1.4	1.9	1	1	1	?
*C. kasignete* Karanovic, 2008	2.3	0.9	1.5	1.9	0.9	P	0.4	10	1	1	5	3/6	6	0/0/0/3	3	8	?	5	1.4	–	1.7	2.1	1.1	1.2	0	+
*C. kasis* Karanovic, 2008	2.4	1.2	2.2	1.6	1.6	P	0.2	9	1	1	5	2/4	6	0/0/3	3	8	?	5	1.5	–	1.5	1.6	1.1	2.1	?	?
*C. kieferi elongata* Herbst, 1953	3.3	1.5	1.7	1.9	1.4	A	0.4	10	0	1	4	3/6	7	0/0/1/4	3	8	?	5	1.6	+	1.8	1.7	1.4	1.5	?	?
*C. kieferi* Schäfer, 1936	1.5	1	2.9	1.8	1.9	P	0.3	?	1	1	5	3/6	7	0/0/1/3	3	8(7)	?	5	1.5	+	1.9	2.1	1.2	2.5	1(0)	?
*C. kiraensis* Hiromi, 1984	3.3	1.2	1	1.4	0.8	A	0.6	10	1	1	5	3/6	7	0/0/1/3	3	8	1.5	5	1.4	+	1.1	1.8	1.5	1.7	2	+
*C. koreana* sp. nov.	3.5	1.6	0.9	1.7	0.9	A	1.4	10	2	1	4	3/6	7	0/0/1/3	3	8	1.6	4	1.7	+	1.3	1.6	1	1.3	2	–
*C. laurentica* Nicholls, 1939	0.5	0.5	8.2	1.4	?	P	0.3	12	0	1	3	3/5	7	0/0/0/4	3	8	1.5	5	1.6	–	1.2	1.9	1	1.5	?	?
*C. mediterranea* Steuer, 1940	1.5	1	2.9	2	1.2	P	0.3	10	1	1	5	3/6	7	0/0/1/3	?	8	1.3	5	1.3	+	1.5	1.8	0.6	1	1	–
*C. norvegica* Boeck, 1865	2.5	1.2	1.1	2.2	2.7	A	0.6	10	?	?	?	?	?	?	?	?	?	?	?	?	1.3	2	1.4	1.8	?	?
*C. oblivia* Monchenko, 1981	2.3	1	1.9	1.6	1.3	P	0.3	10	1	1	5	3/6	6	0/0/1/3	3	8	1.4	5	1.4	–	1.3	1.5	0.7	1	?	?
*C. pacifica* Smirnov, 1935	2.4	1.1	3	1.4	1.5	P	0.3	13	1	1	5	3/6	?	?	3	?	?	?	?	?	1	2	1.2	1.8	?	?
*C. parapsammophila* Monchenko, 1981	1.3	0.8	2.4	3	2.1	P	0.6	10	2	1	4	3/6	7	0/0/1/3	3	8	1.8	4	1.3	–	1	1.7	0.8	1.4	0	+
*C. phoenicia* Lindberg, 1953	3	1	1.5	1.6	1.6	P	0.3	10	1	1	4	3/5	?	?	?	?	?	5	1.4	?	2	2	1.2	1.2	?	?
*C. pontica* Monchenko, 1977	3.7	1.3	0.9	1.6	1.5	A	0.7	10	1	0	4	3/5	7	0/0/1/3	2	8	1.6	5	1.6	–	1.8	1.8	1	1.2	?	?
*C. psammophila* Steuer, 1940	1	0.7	4.7	1.8	1.8	P	0.3	10	0	1	5	?	6	0/0/1/3	3	8	?	4	1.5	–	1	1.7	0.8	1.8	0	?
*C. pygmaea* Sars, 1918	5.5	2	0.8	1.8	2	A	0.8	10	1	1	4	?	?	?	3	7	?	?	?	?	1.1	1.5	1.2	0.9	?	?
*C. rotundipes* Herbst, 1952	2.5	1.2	1	1.4	1.4	A	0.6	10	0	1	4	3/5	6	0/1/2	3	7	1.1	5	1.1	–	1.4	1.3	0.9	1	1	+
*C. schneideri* Scott T., 1903	1.6	0.9	?	?	?	P	0.5	12	1	1	3	3/6	?	?	3	8	?	?	?	?	1	1.5	0.9	1.5	?	?
*C. sinaitica* (Por, 1979)	2.9	1.4	1.3	1.8	1.7	A	0.5	10	1	1	5	3/5	6	1/1/3	2	7	1.3	5	1.6	–	1.3	1.7	1.2	1.4	2	–
*C. smirnovi* Herbst, 1982	1.3	0.9	?	?	?	P	?	?	?	?	?	?	?	?	?	?	?	?	?	?	1.1	1.4	1.5	4.1	1	–
*C. soror* Karanovic, 2008	1.5	0.7	3.4	1.8	1.4	P	0.2	11	1	1	5	3/4	6	0/0/1/3	3	8	?	5	1.7	–	1.9	2.9	0.3	0.6	?	?
*C. steueri* Früchtl, 1923	2.2	1.2	2	1.6	1.9	A	0.5	10	0(1)	0	5	3/6	6(7)	0/0/1/3	3	7	1.5	5	1.5	–	1.5	1.6	1.1	1.1	1	+
*C. tuberculata* Herbst, 1962	3.2	1.8	0.8	1.8	2.1	A	0.2	10	1	1	5	3/6	6	0.1.3	3	?	1.5	5	?	–	1.4	2.5	1.6	1.3	?	?
*C. unisetosa* Karanovic, 2008	2	1	2.2	1.9	1.9	P	0.3	10	1(2)	1	4	3/4	6	0/0/0/3	3	8	1.5	5	1.5	–	1.4	2	1	1.7	?	?
*C. vachoni* Nicholls, 1939	2	1.1	0.7	1.1	?	P	0.2	10	0	1	3	2/6	7	0/0/0/4	3	8	1.5	5	1.6	–	1.1	1.7	0.6	1.2	?	?
*C. wido* sp. nov.	2.3	1.4	0.6	1.2	0.6	A	0.3	10	0	1	4	3/6	6	0/0/1/3	2	7	1.1	5	1.3	–	1.9	1.7	0.5	0.7	2	–
*C. yutimaete* Lotufo, 1994	2.7	1.5	1.5	1.9	1.7	A	0.3	10	2	1	5	3/5	7	0/0/1/4	2	8	1.4	5	1.5	+	1.3	1.8	1.1	1.5	?	?

*Cyclopina koreana* is easily distinguishable from most congeners by its slender and widely spaced caudal rami, as well as by only four setae on the third exopodal segment of fourth leg. Only *C. adelphae* Karanovic, 2008 has somewhat similar caudal rami, but this Australian species has a completely different armature formula of the antennula, antenna, and swimming legs, as well as a more bulbous copulatory duct and slenderer fifth leg. Four setae on the third exopodal segment of fourth leg is a character so far reported only for three other congeners (see Table 1): *C. caroli* Lotufo, 1994 from Brazil; *C. parapsammophila* Monchenko, 1981 from the Black Sea; and *C. psammophila* Steuer, 1940 from the Mediterranean and Red Sea (see Steuer 1940; Herbst 1964; Monchenko 1981; Lotufo 1994). All three, however, have very short caudal rami, and the latter two also have four setae on the third exopodal segment of third leg. Note that Lotufo (1994) stated in his description that *C. caroli* has five setae on the third exopodal segment of the fourth leg, but his fig. 13 clearly shows four and he did not mention this character as variable.

Including *C. curtijeju*, there are currently only seven species of *Cyclopina* with caudal rami that are less than 1.5 × as long as wide (Table 1). Among them the new South Korean species is the only one with an 11-segmented antennula. In fact, they all differ markedly in so many morphological details that it is probably safe to assume that short caudal rami originated convergently in this genus a number of ×. A further 12 species have caudal rami that are between 1.5 and 1.9 × as long as wide (Table 1). Among them, only two have an 11-segmented antennula, both of them Australian endemics: *C. amita* Karanovic, 2008 and *C. soror* Karanovic, 2008. However, they both have no lanceolate setae on the fourth leg endopod, and have only six setae on the maxillular endopod (vs. seven in *C. curtijeju*) and four setae on the second segment of mandibular endopod (vs. six in *C. curtijeju*); additionally, *C. amita* has a different maxillipedal armature formula, while the fifth leg in *C. soror* has the medial spine longer than lateral spine (Karanovic 2008). Most of the other 12 species with relatively short caudal rami have a 10-segmented antennula, except the Brazilian *C. caissara* Lotufo, 1994 and the Scandinavian *C. schneideri* Scott T., 1903, which both have a 12-segmented antennula (see Scott T. 1903; Sars 1913; Lotufo 1994). Note that both Sars (1921) and Gurney (1927) considered *C. brevifurca* Sars, 1913 a subjective junior synonym of *C. schneideri*, presumably because both species were described from Norway and Sars (1913) was not aware of Scott’s (1903) paper, but morphological differences between them are significant enough to consider them as separate species (Table 1). For two *Cyclopina* species with short caudal rami we don’t know the segmentation of female antennula: *C. kieferi* Schäfer, 1936 and *C. smirnovi* Herbst, 1982. The former is presumably widely distributed in Europe (see Schäfer 1936; Steuer 1940; Petkovski 1955) and differs from *C. curtijeju* by antennal armature and proportions of the fifth leg. The latter was proposed as a new name by Herbst (1982) for a single male from Vladivostok, originally identified by Smirnov (1935) as *C. brachystylis* Sars, 1921 and illustrated by two simple drawings. This species could be closely related to *C. curtijeju*, but the lack of information on *C. smirnovi* and the fact that only females were found for the South Korean new species preclude further discussion.

*Cyclopina wido* has a completely unique swimming legs armature formula in the genus. It shares its two-segmented endopod of the first leg with only four congeners: *C. arenosa* Lotufo, 1994 from Brazil; *C. pontica* Monchenko, 1977 from the Black Sea; *C. sinaitica* (Por, 1979) from the Red Sea; and *C. yutimaete* Lotufo, 1994 from Brazil. All these species, however, have two setae on the second endopodal segment of second to fourth legs and differ from *C. wido* in many additional morphological characters (see Monchenko 1977; Por 1979; Lotufo 1994; Table 1). There could be very little doubt that the two-segmented condition evolved in this group convergently. This is further supported by the fact that a two-segmented endopod of the first leg could be found in several unrelated cyclopinid genera (see Herbst 1952, 1964; Krishnaswamy 1957; Plesa 1961; Rao and Ganapati 1969; Herbst 1974; Lotufo and Rocha 1991), and was also once reported as intraspecific variability (Ivanenko and Defaye 2004).

Several problems illustrated above should make it obvious that the genus *Cyclopina* is in need of revision. Unfortunately, as already mentioned by several researchers (Jaume and Boxshall 1996; Ivanenko and Defaye 2004; Karanovic 2008), incomplete descriptions of many species and a lack of one sex in some make this task impossible. To help facilitate further studies in this genus a list of characters is provided below for 48 species and subspecies currently considered as valid (Table 1). It does not include the Chinese *C. heterospina* Shen & Bai, 1956, which appears to have an 18-segmented antennula without elongated sixth segment and a fifth leg exopod with only two elements (see Shen and Bai 1956). This is obviously a completely different genus, but so many morphological details are missing from the species description that it is impossible to postulate phylogenetic relationships with the existing cyclopinid genera. On the other hand, very detailed descriptions and illustrations (including SEM photographs) of the Mexican *Mexiclopina campechana* Suárez-Morales & Almeyda-Artigas, 2015 leave very little double that this is a member of *Cyclopina* in its current (broad) definition, which could be already guessed from a very comprehensive comparison Suárez-Morales and Almeyda-Artigas (2015) provided with *C. esilis* Brian, 1938 and *C. kieferi* Schäfer, 1936. Therefore, it is included in Table 1 as *Cyclopina campechana* (Suárez-Morales & Almeyda-Artigas, 2015) comb. nov. Characters and measurements in this table were scored from original descriptions but also from subsequent redescriptions. Reported variability and/or asymmetries for discrete characters (such as armature formulae) are included in brackets, while those for continuous characters (such as various proportions) were averaged and rounded to the first decimal. The latter are, of course, approximate, which is one of the reasons they should be taken with caution and not used to construct keys to species. In addition to original species descriptions, which are automatically included in the reference list below, and papers already mentioned above, the following publications were consulted for species listed in Table 1: *C. brevifurca* Sars, 1913 (see also Lang 1946); *C. caissara* Lotufo, 1994 (see also Gómez & Martínez Arbizu 2004); *C. ensifera* Grandori, 1926 (see also Brian 1928; Petkovski 1955); *C. mediterranea* Steuer, 1940 (see also Petkovski 1955; Lotufo 1994); *C. norvegica* Boeck, 1865 (see also Sars 1921; Lang 1946); and *C. steueri* Früchtl, 1923 (see also Herbst 1955; Plesa 1963; Monchenko 1976). Armature formula for the maxillipedal endopod is given for the last four segments only. Unfortunately, state of the terminal seta on tip of the exopod of mandibular palp is unknown in most *Cyclopina* species, and therefore is not included in the table. However, there is no doubt that the state of this character would be very important in any phylogenetic analysis; this seta is modified (umbrella-like) in *C. esilis* (see Jaume and Boxshall 1996), as well as in *C. gracilis* and probably several other congeners (D. Jaume, pers. comm. July 2020). All South Korean new species, as well as all Australian species (Karanovic 2008), have this seta unmodified, so any revision of this genus will have to test the significance of this morphological character using molecular tools.

## Supplementary Material

XML Treatment for Cyclopina busanensis

XML Treatment for Cyclopina koreana

XML Treatment for Cyclopina curtijeju

XML Treatment for Cyclopina wido
